# A Novel Class of Small Molecule Agonists with Preference for Human over Mouse TLR4 Activation

**DOI:** 10.1371/journal.pone.0164632

**Published:** 2016-10-13

**Authors:** Jason D. Marshall, Darren S. Heeke, Eileen Rao, Sean K. Maynard, David Hornigold, Christopher McCrae, Neil Fraser, Andrey Tovchigrechko, Li Yu, Nicola Williams, Sarah King, Martin E. Cooper, Adeline M. Hajjar, Jennifer C. Woo

**Affiliations:** 1 Vaccine Platform Group, MedImmune, Gaithersburg, Maryland, United States of America; 2 Translational Biology Group, MedImmune, Mountain View, California, United States of America; 3 Department of Cardiovascular and Metabolic Diseases, MedImmune, Cambridge, United Kingdom; 4 Translational Science, Respiratory, Inflammation and Autoimmunity Innovative Medicines, AstraZeneca R&D, Mölndal, Sweden; 5 Biology Department, AstraZeneca R&D, Charnwood, United Kingdom; 6 Research Bioinformatics, MedImmune, Gaithersburg, Maryland, United States of America; 7 Statistical Sciences, MedImmune, Gaithersburg, Maryland, United States of America; 8 Medicinal Chemistry Department, AstraZeneca R&D, Charnwood, United Kingdom; 9 Department of Comparative Medicine, University of Washington, Seattle, Washington, United States of America; Scripps Research Institute, UNITED STATES

## Abstract

The best-characterized Toll-like receptor 4 (TLR4) ligands are lipopolysaccharide (LPS) and its chemically modified and detoxified variant, monophosphoryl lipid A (MPL). Although both molecules are active for human TLR4, they demonstrate a potency preference for mouse TLR4 based on data from transfected cell lines and primary cells of both species. After a high throughput screening process of small molecule libraries, we have discovered a new class of TLR4 agonist with a species preference profile differing from MPL. Products of the 4-component Ugi synthesis reaction were demonstrated to potently trigger human TLR4-transfected HEK cells but not mouse TLR4, although inclusion of the human MD2 with mTLR4 was able to partially recover activity. Co-expression of CD14 was not required for optimal activity of Ugi compounds on transfected cells, as it is for LPS. The species preference profile for the panel of Ugi compounds was found to be strongly active for human and cynomolgus monkey primary cells, with reduced but still substantial activity for most Ugi compounds on guinea pig cells. Mouse, rat, rabbit, ferret, and cotton rat cells displayed little or no activity when exposed to Ugi compounds. However, engineering the human versions of TLR4 and MD2 to be expressed in mTLR4/MD2 deficient mice allowed for robust activity by Ugi compounds both in vitro and in vivo. These findings extend the range of compounds available for development as agonists of TLR4 and identify novel molecules which reverse the TLR4 triggering preference of MPL for mouse TLR4 over human TLR4. Such compounds may be amenable to formulation as more potent human-specific TLR4L-based adjuvants than typical MPL-based adjuvants.

## Introduction

The innate immune system has evolved to recognize common pathogen-derived molecular patterns (PAMPs) with sentinel molecules known as pattern recognition receptors (PRRs). The most well-studied family of PRRs are the Toll-like receptors (TLRs), which are notably expressed by several types of antigen-presenting cells (APCs), including dendritic cells, macrophages, B cells, and keratinocytes. Some members of the TLR family are expressed on the cell surface where they are activated largely by bacterially derived ligands such as cell wall components, lipopeptides, and flagellin. Other TLRs are displayed intracellularly within the context of the endosomal system of degradative compartments, where they primarily sample RNA and DNA fragments derived from degraded viral particles.

Among the better characterized of the TLR family is TLR4 which is singular among TLRs in several ways. TLR4 requires the accessory molecule MD2 and activates two distinct signaling pathways mediated by the adapter proteins, myeloid differentiation primary response 88 (MyD88) and TIR domain-containing adaptor inducing interferon-beta (TRIF), while most other TLR family members do not associate with accessory molecules and signal through one main pathway [1]. TLR4 appears to accomplish this by signaling through MyD88 when recognizing ligands at the cell surface but then switching to TRIF signaling once internalization of TLR4 has occurred [2], [3]. MyD88-mediated signaling from the cell surface triggers the translocation of NFkB to the nucleus and consequent activation of pro-inflammatory cytokines, while the TRIF pathway leads to the phosphorylation of IRF transcription factors and expression of genes from the type I interferon family.

A broad range of exogenous and endogenous ligands are recognized by TLR4, from polysaccharides to lipid-containing molecules to peptides and proteins [4]. The classical and first-discovered TLR4L (TLR4 ligand) is LPS, a structural component of the outer membrane of most Gram-negative bacteria [5]. LPS is composed of a hydrophobic lipid A domain which can intercalate with the plasma membrane and three sugar domains: an inner core oligosaccharide chain, an outer core oligosaccharide, and O-antigenic polysaccharide. Mild acid hydrolysis of LPS from Salmonella minnesota R595 results in a detoxified form of the lipid A portion, MPL, which has earned a favorable safety profile due to its reduced ability to induce proinflammatory cytokines, eventually resulting in its approval by the FDA for use as the first TLR-based adjuvant in humans as a component of AS04, in which MPL is formulated with aluminum salts. AS04 has been approved for use in the vaccines Fendrix and Cervarix.

Although proven to be active in humans, MPL has some suboptimal qualities, primary of which is its heterogeneous nature as a mixture of tetra-, penta-, and hexa-acylated lipids, which can vary widely in acyl chain length and in potency. Tetra-acylated species, in particular, generally have little or no human TLR4-triggering activity and can even act as antagonists [6]. In addition, although MPL has an extremely reduced reactogenicity profile in toxicity studies, its immunostimulatory profile is also much reduced in comparison to LPS [7]. In an attempt to generate a purer and more potent TLR4L preparation, several synthetic lipid A analogs have been developed as adjuvants for clinical use, including aminoalkyl glucosaminide 4-phosphates (AGPs), glucopyranosyl lipid A (GLA) [7], eritoran [8], and the simplified lipid A mimetic engineered without a disaccharide backbone, E6020 [9], [10]. As reports have indicated that appropriate formulation of lipid A analogs with an adjuvant delivery system substrate like alum, liposomes, or squalene-based oil-in-water emulsions [11] can greatly amplify TLR4-directed immune activity, several lines of research have focused on engineering potent TLR4-based adjuvants that maximize immunostimulatory activity on TLR4^+^ target cells, while dose-sparing allows for minimization of adverse effects. One of the most promising of this new generation of adjuvants is GLA/SE [12], in which the rationally designed lipid A analog GLA has been physically incorporated into the squalene oil phase of a squalene emulsion. This manner of presentation of the TLR4L results in a substantial increase in adjuvant activity per GLA molecule compared to aqueous-formulated or alum-formulated GLA [13].

Because of their amphiphilic nature and solubility profile, lipid A analogs are ready-made for addition to lipid- and oil-based substrates. However, their chemical nature also makes such compounds more difficult to work with when formulating them with nanoparticles that prefer water-soluble agents for encapsulation. In this paper, we present the discovery of a new class of small molecule agonist for the TLR4 receptor that is structurally and chemically distinct from previously reported TLR4 agonists. These compounds, synthesized through the 4-component condensation reaction known as the Ugi reaction, were found to exert potent TLR4-triggering activity of both hTLR4-transfected HEK293 cells and human PBMCs yet were poorly inductive of mouse TLR4 activity in comparable cell substrates.

## Materials and Methods

### Ethics Statement

Animal studies were conducted under protocols approved by the MedImmune Institutional Animal Care and Use Committee and the Valley Biosystems Institutional Animal Care and Use Committee.

### Synthesis of Ugi compounds

The general procedure for synthesis of the compounds, herein referred to as the Ugi compounds, was as described below for the synthesis of AZ126 and AZ368:

N-(2-(cyclopentylamino)-2-oxo-1-(pyridin-4-yl)ethyl)-N-(4-methoxyphenyl)-3-methyl-5-phenyl-1H-pyrrole-2-carboxamide (AZ126)

Isocyanocyclopentane (6.64 μL, 0.06 mmol) was added to 4-methoxyaniline (7.96 mg, 0.06 mmol), isonicotinaldehyde (6.17 μL, 0.06 mmol) and 3-methyl-5-phenyl-1H-pyrrole-2-carboxylic acid (13 mg, 0.06 mmol) in methanol (3 mL) at 25°C. The resulting solution was stirred at 25°C for 24 hours then at 45°C for 4 days. The reaction mixture was evaporated to dryness and the residue purified by preparative HPLC on a Phenomenex Gemini column using a 75–5% gradient of aqueous 0.2% ammonia in acetonitrile as eluent. The fractions containing the desired compound were evaporated to dryness *in vacuo* to afford the title compound (10.00 mg, 30.4%) as a white solid.

^1^H NMR (400 MHz, CDCl_3_) δ 1.37–1.49 (m, 4H), 1.98–2.04 (m, 4H), 2.31 (s, 3H), 3.80 (s, 3H), 4.24–4.29 (m, 1H), 6.11 (s, 1H), 6.22 (2, 1H), 6.58 (d, 1H), 6.86 (d, 2H), 7.01 (d, 2H), 7.18 (t, 2H), 7.25 (d, 2H), 7.43 (t, 1H), 7.55 (d, 2H), 8.51 (d, 2H).

(E)-3-(4-(2-(cyclopentylamino)-1-(N-(4-isopropylphenyl)-1,5-diphenyl-1H-pyrazole-3-carboxamido)-2-oxoethyl)phenyl)acrylic acid (AZ368)

(E)-3-(4-formylphenyl)acrylic acid (100 mg, 0.57 mmol), 4-isopropylaniline (0.078 mL, 0.57 mmol) and isocyanocyclopentane (0.063 mL, 0.57 mmol) were added to 1,5-diphenyl-1H-pyrazole-3-carboxylic acid (150 mg, 0.57 mmol) in methanol (3 mL) and THF (3 mL) at room temperature. The resulting solution was stirred for 24 hours then the crude reaction mixture was purified directly by preparative HPLC on a Phenomenex Gemini column using a 50–5% gradient of aqueous 0.1% formic acid in acetonitrile as eluent. The fractions containing the desired compound were evaporated *in vacuo* to dryness to afford the title compound (84 mg, 22.67%) as a white solid.

1H NMR (400 MHz, DMSO) δ1.10 (d, 6H), 1.20–1.60 (m, 6H), 1.70–1.82 (m, 2H), 2.78 (7^et^, 1H), 4.00–4–05 (m, 1H), 6.15 (s, br, 1H), 6.26 (s, br, 1H), 6.45 (d, 1H), 6.90–7.15 (m, H), 7.25–7.33 (m, 5H), 7.45–7.52 (m, 3H), 8.14 (d, 1H).

The acidic compounds AZ617, AZ618, AZ161, AZ839, AZ226, AZ635, and AZ902 were synthesized at larger scale following the procedure described below for AZ618, by incorporation of the appropriate amine and isocyanate in the 4-component Ugi reaction step.

1,5-Diphenyl-1H-pyrazole-3-carboxylic acid

Phenylhydrazine (10.81 mL, 108.98 mmol) was added dropwise over 15 minutes to a stirred solution of (Z)-ethyl 2-hydroxy-4-oxo-4-phenylbut-2-enoate (20 g, 90.82 mmol) in ethanol (400 mL) at room temp. When the addition was complete, the mixture was heated to reflux for 3 hours then cooled to room temperature and evaporated in vacuo. The residue was subjected to column chromatography over silica (100g cartridge), eluting with a 5–10% ethyl acetate gradient in heptane. Fractions containing the main regioisomer were combined and concentrated under reduced pressure to a volume of ca. 200ml whereupon formation of a precipitate was observed. After standing at room temperature for 16 hours, the resulting precipitated solid was collected by filtration, washed with heptane and dried in vacuo to give ethyl 1,5-diphenyl-1H-pyrazole-3-carboxylate (21.3 g, 72.86 mmol). This solid was dissolved in THF (250 mL) then ethanol (100 mL) and 2 M sodium hydroxide solution (91 mL, 181.64 mmol) were added. The resulting homogeneous solution was maintained at room temperature for 24 hours then acidified to pH<2, by dropwise addition of conc hydrochloric acid, and extracted with ethyl acetate (2x500ml). Combined organic extracts were dried over anhydrous magnesium sulfate, filtered and evaporated *in vacuo* to give the title compound (18.40g, 69.62mmol, 77%) as a white solid.

1H NMR (500 MHz, DMSO) δ 7.07 (s, 1H), 7.22–7.28 (m, 2H), 7.29–7.39 (m, 5H), 7.41–7.49 (m, 3H), 12.98 (s, 1H).

Methyl 2-(4-formylphenyl)acetate

2-(4-(Bromomethyl)phenyl)acetic acid (25g, 109.14mmol) was added portion wise over 30 minutes to a rapidly stirred solution of copper(II) nitrate trihydrate (39.6g, 163.71mmol) in water (250mL) at 100°C. The resulting suspension was stirred for a further 3 hours at 100°C during which time the solid dissolved completely and a color change from blue to green was observed. The solution was allowed to cool to room temperature and maintained at this without stirring for 16 hours. The resulting precipitated solid was collected by filtration, washed with water (3x20ml) and dried in vacuo to give 2-(4-formylphenyl)acetic acid (13.16g, 80.17mmol). This solid was dissolved in methanol (117ml), the resulting solution cooled to 0°C and stirred during portion wise addition of sulfurous dichloride (2.57ml, 35.25mmol) over 2 hours. The mixture was stirred at 0°C for a further 3 hours then evaporated *in vacuo* (rotary evaporator) with a bath temp of 20°C. The residual oil was added to saturated sodium bicarbonate solution (60ml), the mixture diluted with water (30ml) and acetonitrile (30ml) then extracted with ethyl acetate (200ml + 100ml). Combined organic extracts were washed with brine (60ml), dried over anhydrous magnesium sulfate, filtered and evaporated *in vacuo* to give the title compound (15.67g, 76.72mmol, 70%) as a low-melting solid.

1H NMR (500 MHz, CDCl_3_) δ 3.52 (d, 5H), 7.25 (t, 2H), 7.66 (d, 2H), 9.81 (s, 1H).

2-(4-(2-(cyclopentylamino)-1-(N-(3,5-dimethylphenyl)-1,5-diphenyl-1H-pyrazole-3-carboxamido)-2-oxoethyl)phenyl)acetic acid (AZ618)

A solution of 3,5-dimethylaniline (15.17mL, 121.5mmol) in methanol (50ml) was added dropwise over 1 hour to a stirred suspension of 1,5-diphenyl-1H-pyrazole-3-carboxylic acid (32.1g, 121.5mmol), methyl 2-(4-formylphenyl)acetate (21.64g, 121.5mmol) and isocyanocyclopentane (13.44mL, 121.5mmol) in methanol (700ml) at room temperature. Most of the 1,5-diphenyl-1H-pyrazole-3-carboxylic acid had dissolved before completion of addition. The mixture was stirred for a further 2 hours during which time all remaining solid dissolved. The mixture was evaporated *in vacuo* and the residual oil dissolved in a minimum volume of dichloromethane then diluted with an equivalent volume of heptane (ca. 300ml in total). The resulting solution was applied to a 350g silica cartridge which was eluted with 30% ethyl acetate in heptane. Product-containing fractions were combined and concentrated under reduced pressure to a volume of ca. 500 ml whereupon a thick suspension was obtained. The resulting solid product was collected by filtration, washed with 10% ethyl acetate in heptane (3x100ml) and dried *in vacuo* at 40°C to give methyl 2-(4-(2-(cyclopentylamino)-1-(N-(3,5-dimethylphenyl)-1,5-diphenyl-1H-pyrazole-3-carboxamido)-2-oxoethyl)phenyl)acetate (53.5g, 83.49mmol). This solid was dissolved in a mixture of tetrahydrofuran (600ml) and methanol (240ml) then 2M sodium hydroxide solution (124ml, 248.1mmol) was added to give a homogeneous solution. After 4 hours at room temperature the mixture was concentrated under reduced pressure to ca. 50% of original volume, diluted with water (600ml), acidified with 2M hydrochloric acid to pH<2 and extracted with ethyl acetate (2x600ml). Combined organic extracts were dried over anhydrous magnesium sulfate, filtered and evaporated *in vacuo*. The residual sticky solid was triturated with TBME (500ml), the resulting solid filtered, washed with TBME (3x50ml) and dried *in vacuo* at 40°C to give the title compound (51.0g, 81.37mmol, 67%) as a white powder.

1H NMR (500 MHz, DMSO) δ 1.27 (tt, 1H), 1.33–1.64 (m, 5H), 1.69–1.84 (m, 2H), 1.96–2.14 (m, 6H), 3.47 (s, 2H), 3.95–4.1 (m, 1H), 6.20 (s, 2H), 6.74 (s, 2H), 6.92–7.14 (m, 8H), 7.24–7.42 (m, 6H), 8.07 (d, 1H), 12.27 (s, 1H).

### Endotoxin measurement

Ugi compounds were tested for endotoxin with Endosafe Cartridge Reader by The Endosafe-PTS cartridge (lot# 3305166) after 1:1000 dilution by endotoxin-free water. All compounds were found to have less than 10 EU/ml (< 0.01 EU/μg) of endotoxin.

### Generation of mouse/human TLR4-HEK transfectants

HEK293 cells stably transfected with pNiFty2-SEAP reporter plasmid were transiently transfected with either human or mouse TLR4 (pUNO expression vector), in combination with either human or mouse CD14/MD2 (pDUO expression vector). Transient transfectants were seeded in Dulbecco’s Modified Eagle Medium, FCS 10% (v/v), 2 mM l glutamine and non-essential amino acids.

### Activation of TLR4-HEK transfectants

Cells were seeded in tissue culture treated clear flat bottom polystyrene 96 well plates (Costar, 3598) at 1e4 cells/well. Concentration-response curves were generated by addition of test compounds and incubation for 20 h at 37°C in an atmosphere of 5% CO_2_. LPS (E. coli 0111:B4, Ultra-pure, Invivogen) or PHAD (Avanti Polar Lipids) was used as a positive control. The SEAP released was quantified using p nitrophenyl phosphate as a substrate, and the absorbance at 405 nm was measured at 30 second intervals over several minutes using a microplate reader. Some data are recorded as the pEC50, the negative log of the EC50 value. Other data generated from these reporter cell lines were converted to AUC (area under the curve) values by interpolation of fitted cubic spline curve between OD value and log concentration levels. The calculation is performed using MESS package in statistical software R (www.r-project.org/).

### Generation of guinea pig TLR4-HEK transfectants

Fragments of guinea pig TLR4 were identified by searching the Ensembl genome browser database (http://www.ensembl.org/index.html) using the BLAST algorithm and discontiguous mega blast of the NCBI trace archives (http://www.ncbi.nlm.nih.gov/blast/tracemb.shtml) using the DNA sequence of human TLR4 Isotype A as the search string. Guinea pig CD14 was assembled in silico from fragments identified in the NCBI trace archives using human CD14 as the search string (76% similar/72% identical). Fragments corresponding to guinea pig MD2 were identified by searching the Ensembl genome browser database and NCBI trace archives with human MD2 as the search string (72% similar/68% identical). Sequence information was used to design primers for PCR amplification of full length coding sequences from guinea pig spleen cDNA. Sequences for guinea pig MD2 and CD14 were as predicted in silico. gpTLR4 was cloned into the expression vector pUNO (Invivogen). gpMD2 and gpCD14 were cloned together into pDUO (Invivogen) for co-expression. gpMD2 was also cloned into pUNO for transient co-expression with gpTLR4. A stable mixed population expressing gpTLR4 /CD14/MD2 was made by transfecting the gpTLR4/pUNO and gpMD2 + gpCD14/pDUO constructs into a HEK293 cell-line containing a stably integrated pNiFty2-SEAP reporter plasmid (Invivogen) using Fugene-6 (Roche). The mixed population was selected using the antibiotics blasticidin (pUNO), hygromycin (pDUO) and zeocin (pNiFty2-SEAP) and low passage cells were cryopreserved at -80C in cell freezing mix (80% FCS/10% DMEM/10% DMSO) at a cell density of 6e6 cells/ml. For transient expression, gpTLR4 and gpMD2 cloned independently into pUNO were co-transfected into the HEK293 pNiFty2-SEAP reporter cell-line using Fugene-6. 24 hours post transfection cells were harvested and cryopreserved.

### Guinea-pig TLR4 reporter agonist assay

SEAP detection assay was performed as previous described by [14]. In brief, cryopreserved cells were rapidly thawed and resuspended in DMEM (Gibco) supplemented with 1% (v/v) fetal bovine serum (Sigma Aldrich) and 2 mM glutamine (Sigma Aldrich). 3e5 cells/ ml were seeded into 384 well microtiter plates (Greiner) containing various concentrations of agonist test compounds. Cells and compounds were incubated for 20 h at 37°C in a 5% (v/v) CO2/air environment, 95% humidity. 25 μl of 9 μM DDAO phosphate in DMEM was then added and the plate subsequently incubated at RT for a further 30 min to allow the SEAP to catalyze the DDAO phosphate to the fluorescent product DDAO. Plates were read using the Envision plate reader excitation λex 620nm, emission λem 665nm. Data was analyzed as % maximum 1 μM Ultrapure (0111:B4) LPS activation signal (Invivogen).

### Primary cell isolation

Splenocytes were isolated from naïve BALB/c mice by dissociating spleens with a GentleMACS (Miltenyi) instrument, then filtration through 70 μM screens. For other species, blood was obtained through venipuncture or cardiac puncture into heparinized tubes, then diluted 1:1 with PBS or HBSS and layered onto Ficoll-Histopaque (GE Healthcare) for blood from healthy human volunteers and cynomolgus macaques, Lympholyte-Mammal (Cedarlane Labs) for New Zealand white rabbits, Sprague-Dawley rats, Hartley guinea pigs, cotton rats, and ferrets, followed by standard density centrifugation techniques. PBMCs layers were recovered and cultured in RPMI-1640 + human AB serum (Life Technologies) + Pen Strep + L-Glutamine (human cells) or RPMI-1640 + 10% FBS (Hyclone) + Pen/Strep + L-Glutamine + 0.1% Beta-Mercapto-ethanol (Sigma) (mouse, cyno, rat, cotton rat, guinea pig, rabbit, ferret cells). Purchase of peripheral blood from cynomolgus macaques was from Valley Biosystems (West Sacramento, CA) and was performed in accordance with Standard Operating Procedures and with the approval of the Valley Biosystems Institutional Animal Care and Use Committee (IACUC). Valley Biosystems is fully accredited by the Association for the Assessment and Accreditation of Laboratory Animal Care International. Animals were immobilized with a single intramuscular injection of ketamine (10 mg/kg). Following sample collection, the animals were returned to their home cages. Immobilization lasted for approximately 20 minutes and the animals slowly recovered without incident. No cynomolgus macaques were euthanized to collect the data presented in this study. Collection of peripheral blood from healthy human volunteers was reviewed and approved by the Chesapeake Institutional Review Board (Columbia, MD). Collection of blood and lymphoid tissues from mice, rats, cotton rats, guinea pigs, ferrets, and rabbits was reviewed and approved by the Institutional Animal Care and Use Committee (IACUC) of MedImmune.

### Analysis of cytokine induction by Luminex and QuantiGene

PBMCs (human, cynomolgus macaque, guinea pig, ferret, rabbit, rat, and cotton rat) or splenocytes (mouse) were seeded onto 96-well flat bottom plates at 5 x 10^5^ cells/well and stimulated with TLR4 agonists at 1 and 0.2 μg/ml for 24 h at 37°C. MPL (monophosphoryl lipid A from *Salmonella minnesota* R595) and LPS EK (E.coli K12) were procured from InVivogen. For human, cyno, mouse, and rat, cell-free SNs were analyzed via human, NHP, mouse, and rat MILLIPLEX kits. For GP, ferret, rabbit, and CR, cells were lysed by Millipore lysing buffer and snap frozen at -70 C until ready for analysis by Affymetrix QuantiGene 2.0 Plex kits. The steps of target hybridization, signal amplification, and detection were performed according to manufacturer’s protocol. Signal was detected using a Bioplex 200 instrument (BioRad). Since no one chemokine analyte was available in every one of the 8 species-specific assays, data were collected for any available analyte of three different chemokines, MCP-1, MIP-1β, and RANTES.

### Generation and in vivo use of hTLR4/MD2 transgenic mice

The humanized TLR4/MD-2 mice, described in detail in [15], express human TLR4 and human MD-2 from bacterial artificial chromosome transgenes that were bred to the mouse TLR4/MD-2 double-knockout (DKO) on a C57BL/6J background. Because the hMD-2 BAC transgene is integrated on the Y-chromosome, only male mice were used in the study. Generally, hMD-2 BAC+ males are bred to hTLR4 BAC+ females (both on DKO background) and male pups were screened for the presence of hTLR4 BAC. Double positives served as the fully humanized TLR4/MD-2 mice and hTLR4 BAC negative mice served as functional KO. Wild-type C57BL/6 mice were bred in parallel. All breeding was performed in an SPF facility at the University of Washington and all protocols were approved by the University of Washington IACUC. For in vivo cytokines, mice were injected intraperitoneally with 50 μg of either AZ618 or MPL in 0.2 ml PBS. Mice were bled from the retroorbital sinus 2 h after injection and terminal cardiocentesis was performed at 6 h for the second blood sample. Serum samples were analyzed via MILLIPLEX kits (EMD Millipore).

### Derivation and stimulation of mouse BMDCs

Femur/tibia bones from w.t. C57BL/6, TLR4/MD2 KO, and hTLR4/MD2 KIKO mice were soaked in 10% FBS-RPMI. Muscle and fat tissue was stripped off from the bones with a scalpel, then, bone pairs from individual mice were transferred to 100 mm petri dishes. Femur and tibia bones were separated and the ends cut with dissecting scissors so that bone marrow cells (BMCs) could be removed by flushing the interior of the bones using a 21 G needle and warm 10% FBS-RPMI. BMCs were passed through a 70 μm strainer, followed by RBC lysis. BMCs were cultured in a 70 mm flask for 24 h in the presence of 10 ng/ml (200 unit/ml) of mGM-CSF. Non-adherent cells were collected, washed, and seeded in a 175 cm flask in the presence of 20 ng/ml of mGM-CSF. Every two days, the cells were fed with fresh media and 20 ng/ml mGM-CSF. On day 9, cells were harvested, washed, and stained for CD11c/CD11b expression to determine BMDC density. BMDCs were cultured at 50,000 cells/well for 24 h with 1 μg/ml MPL, LPS, Ugi compounds, or 2 μM R848 (InVivogen).

### Bioinformatics analysis

All orthologous proteins for Ly96 (MD2) were downloaded from GenBank (http://www.ncbi.nlm.nih.gov/gene/?term=ortholog_gene_23643%5Bgroup%5D). In order to select one sequence per species among multiple isoforms, we picked those that had highest coverage when aligned against the available representatives from the Protein Data Bank (PDB) [16] preferring sequences from the RefSeq collection if there were still multiple candidates per species. Multiple Sequence Alignment (MSA) was generated with MAFFT [17].

All macromolecular structural visualizations were generated with PyMol v. 1.7.6 [The PyMOL Molecular Graphics System, Version 1.8 Schrödinger, LLC]. 3D structures of the ligands for small molecule docking were generated by Avogadro [18] from SMILES strings and subjected to 4000 rounds of minimization with GAFF forcefield in OpenBabel [19]. Docking was performed with Smina [20], a version of AutoDock Vina [21]. TLR4/MD2 structures from PDB entries 3VQ2 [22] (mouse) and 4G8A [23] (human) were used to represent receptor in a homodimer of heterodimers conformation induced by binding of TLR4/MD2 to a natural LPS ligand. PDB entry 5IJB [24] (mouse) and 5E56 [http://dx.doi.org/10.2210/pdb5e56/pdb] (human) were used for the receptor in unbound ligand-free conformation. Because 5E56 contained only MD2 protein without the TLR4 (the only available PDB structure for human MD2), we added TLR4 from 4G8A complex after structural superposition of MD2 between 4G8A and 5E56. Chains A (for TLR4), C (for MD2) and B (for the second TLR4 from the dimerized receptor) were used for docking from the biounit files provided by the PDB. During docking, a shell of flexible receptor residues was defined as follows: any residue in chains A or C with any atom within 3.4 Å from either of the native human or mouse LPS ligands after superimposing human and mouse receptors into a best fit orientation. For chain B, the distance cutoff was for the flexible residue selection was 4.0 Å. The search region for the docking was defined as a box enclosing LPS ligand extended by 6 Å in every direction.

Each of the Ugi compounds AZ161, AZ617, AZ618, AZ839 and AZ902 was docked independently. Docking was performed with 48 independent trajectories for each run (parameter—exhaustiveness of Smina). To evaluate convergence, we also performed docking with 96 and 144 trajectories for a subset of simulation runs, and obtained qualitatively identical results. Predicted ligand positions representing nine top-ranked clusters of conformations were saved from each run.

PDB structure 5IJC [24] was used to compare our predicted ligand binding modes with those observed for Neoseptin-3 peptidomimetic compound.

## Results

### Discovery and optimization of Ugi compounds

**Fig 1:**
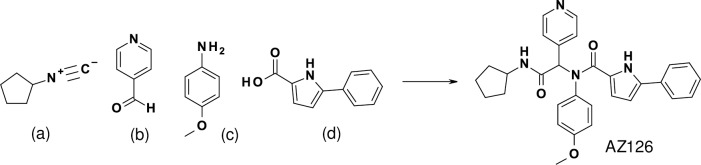
Ugi coupling reaction. The Ugi coupling reaction involves the combination of isocyanide (a), aldehyde (b), amine (c) and carboxylic acid (d) components in a single reaction step to give an α-aminoacyl amide product, as exemplified above for the preparation of AZ126.

A high-throughput search for small molecule TLR4 agonists using an NFκB SEAP reporter gene assay in hTLR4-transfected HEK293 cells yielded the discovery of the selective full agonist AZ126 with a pEC_50_ value of 5.6. AZ126 was the product of a 4-component “Ugi” coupling reaction of an isocyanide, an aldehyde, an amine and a carboxylic acid (Fig 1).

**Fig 2:**
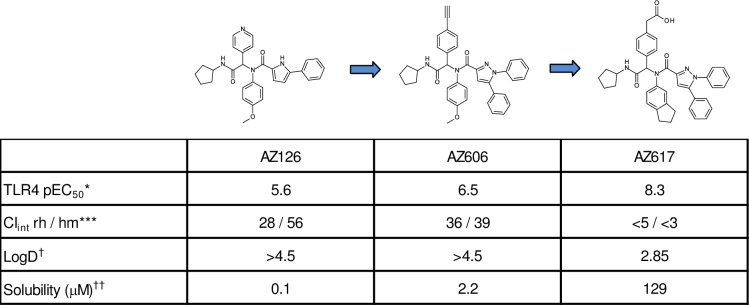
Optimization stages of Ugi products as TLR4 agonists. *Human TLR-4 NFκB SEAP reporter assays; protocol as described in Methods. Data are reported as pEC50, or negative log of the EC50 values. **Cl_int_ rh / hm represents intrinsic clearance in presence of rat hepatocytes (μL/min/10^6^ cells) and human microsomes (μL/min/mg protein). As a general guide, a value of <10 indicates good metabolic stability while >30 suggests that the compound is likely to be rapidly metabolized in vivo. ^†^LogD represents the lipophilicity, whereby D is the octanol/water distribution coefficient at pH7.4. Thus a drug molecule with LogD >4 might be considered to be very lipophilic and therefore more likely to suffer from rapid metabolic turnover, poor solubility and receptor promiscuity; whereas a LogD range of 1–3 is more likely to be associated with optimal physicochemical and drug-like properties. ^††^Solubility was measured by dissolution of solid samples in buffer at pH 7.4.

Although AZ126 was lipophilic and only exhibited modest activity in the hTLR4-HEK assay, rapid optimization was possible by variation of the Ugi components using a combinatorial chemistry strategy. Such an approach initially yielded AZ606, which incorporated a diphenyl imidazole moiety and resulted in a 10-fold increase in potency. Subsequent incorporation of a carboxylic acid group, as in AZ617, led to a further significant increase in potency (Fig 2). Additionally, the reduced lipophilicity (LogD) afforded by the acid group was accompanied by greatly improved metabolic stability and aqueous solubility of AZ617, thus potentially making compounds from this series suitable for oral dosing (Fig 2). Furthermore, whilst many of the earlier-stage Ugi compounds that were more lipophilic were also found to be potent inhibitors of one or more cytochrome P450 enzymes, this activity appeared to have been greatly reduced in AZ606 and abolished in AZ617 (unpublished data).

**Fig 3:**
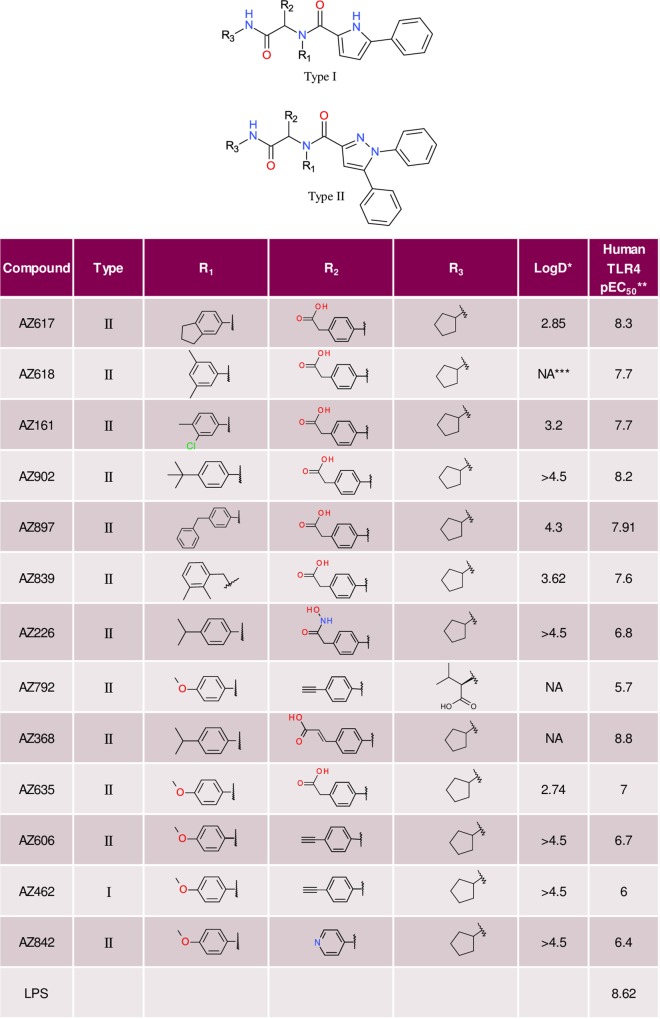
Ugi reaction products with variation of side-chains. *LogD represents the lipophilicity whereby D is the octanol/water distribution coefficient at pH7.4; whereby LogD 1–3 is considered optimal for drug-like properties. **Human TLR-4 NFκB SEAP reporter assay; protocol as described in Methods (N.B. pEC_50_ = –Log_10_(EC_50_), thus values of 6 and 9 represent EC_50_ values of 1 μM and 1 nM (0.001 μM) respectively. ***NA = not available.

With an improved in vitro profile, AZ617 and several additional analogs incorporating the diphenyl imidazole moiety and embodying a range of lipophilicity from side-chain variation were synthesized as candidates for activity characterization (Fig 3). Most of these compounds were found to have potent TLR4-triggering activity of human TLR4-transfected HEK293 cells, and several approached the activating potential of LPS. All Ugi compound preparations were tested for the presence of endotoxin and found to be negative (Methods).

### Ugi compounds trigger TLR4 activity in hTLR4-HEK but not mTLR4-HEK transfectants

**Fig 4:**
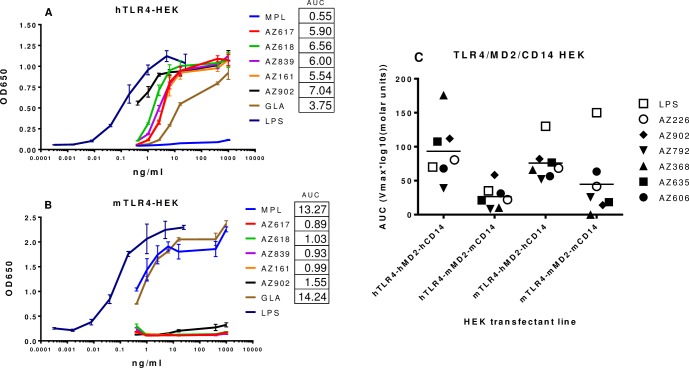
Ugi compounds preferentially activate TLR4-HEK transfectants expressing human MD2. A-B) HEK293 cells transfected with human TLR4/MD2 or mouse TLR4/MD2 (InVivogen) were stimulated with TLR4 agonists including MPL, PHAD, and Ugi compounds at 1 μg/ml to 4 ng/ml and with LPS at 25 ng/ml to 64 pg/ml for 24 h. SNs were analyzed for SEAP content by the QUANTI-Blue assay (InVivogen) and analysis at 650 nm by spectrophotometer. AUC (area under the curve) values were calculated as in Methods. No AUC value was calculated for LPS since it was utilized with a different dose range. C) HEK transfectants with cis- or trans-species expression of TLR4, MD2, and CD14 were stimulated with Ugi compounds in a dose response range of 9 μM to 1 nM or with LPS (boxes) at 75 ng/ml to 5 pg/ml for 48 h. Data was obtained as Vmax calculated over the initial linear portion of the kinetic absorbance measurements. The AUC (area under the curve) value for the dose response curve of Vmax vs log_10_ (molar units) was calculated for each compound using Trapezoidal rule.

Although no activity was found when Ugi compounds were used to stimulate transfectants expressing human TLR2, TLR7, or TLR9 (S1 Fig), hTLR4/hMD2 transfectants were stimulated by these compounds in robust fashion with AUC values higher than the traditional TLR4 agonists MPL and PHAD (Fig 4A). Interestingly, these same compounds were observed to have substantially lower activity on mTLR4/mMD2 transfectants (Fig 4B), unlike MPL and PHAD, which have been found to be active on nearly all species [1]. Additionally, we verified that Ugi compounds exert no activity on hTLR4-HEK transfectants without the presence of hMD2 but do not require the presence of hCD14 (S2 Fig).

Since Ugi compounds displayed a species preference for triggering hTLR4 transfectants and not mTLR4 transfectants, we generated a series of new HEK293 transfectants that would allow determination of which components of the TLR4 signaling complex were integral for signaling by Ugi compounds. Transient transfection of HEK293 cells generated populations expressing cross-species combinations of TLR4 and MD2: hTLR4/mMD2/mCD14 and mTLR4/hMD2/hCD14. After stimulation with a titration of LPS, the two lines that expressed mTLR4 exhibited the highest levels of activity (Fig 4C). This coincides with observations that the TLR4 component of the signaling complex is more important than MD2 for optimal triggering by LPS, and that murine TLR4 can recognize some LPS variants not recognized by the human TLR4 complex [25]. However, most Ugi compounds induced robust activity from the two lines that expressed human MD2 but substantially lower activity from both mMD2-expressing lines (Fig 4C). This suggests that the presence of human MD2 expressed by HEK293 transfectants was preferred for optimal triggering by Ugi compounds but that the species origin of TLR4 was a lower impacting factor.

### Ugi compounds interact with the TLR4 signaling complex in a fashion distinct from LPS

**Fig 5:**
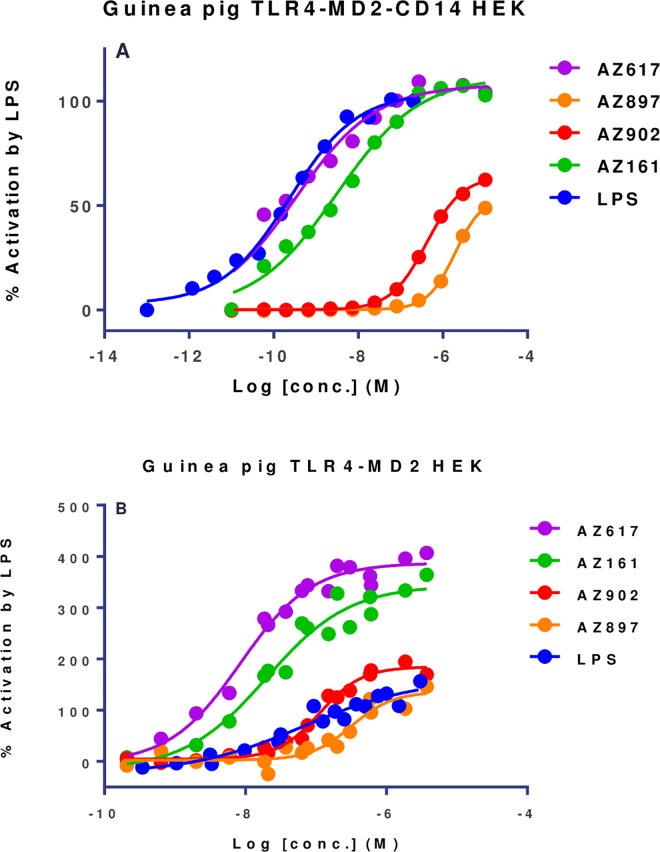
Ugi compounds activate guinea pig TLR4/MD2-HEK transfectants. HEK293 cells underwent transient co-transfection with expression vectors for guinea pig TLR4, MD2, and CD14 along with SEAP reporter plasmid. Transfectants were stimulated with TLR4 agonist compounds at 10^−5^ to 10^−10^ M for 20 h. The plates were developed with the addition of DDAO phosphate as substrate and then read on a plate reader at 620 nm. Data were analyzed as % of the maximum 1 μM Ultrapure (0111:B4) LPS activation signal.

In order to test species specificity with a third transfectant, guinea pig TLR4, MD2, and CD14 were cloned, inserted into vectors and transiently transfected into HEK293 cells. gpTLR4/gpMD2/gpCD14 HEK293 transfectants responded vigorously to LPS stimulation as did mouse and human TLR4 transfectants (Fig 5A). The family of Ugi compounds also demonstrated a range of potencies that indicated some compounds such as AZ617 and AZ161 had potency comparable to LPS while others like AZ902 were less active but still positive.

LPS also requires the presence of CD14 for optimal activity, as CD14 plays the role of transferring LPS from the soluble chaperone molecule LBP to the TLR4 signaling complex [26]. When gpCD14 was left out of the transient transfection, the ability of LPS to trigger the gpTLR4 complex was much reduced (Fig 5B). In contrast, all of the Ugi compounds tested retained their potency in the absence of gpCD14, demonstrating a lack of a requirement for contribution of this molecule for optimal triggering of the TLR4 complex. This observation was subsequently confirmed with hTLR4/hMD2 transfectants that lacked hCD14 (data not shown).

### Ugi compounds show species specificity preference for human and guinea pig in primary cells

In order to explore more completely the issue of species specificity of the Ugi compounds and to select an animal model in which to conduct in vivo studies, we tested the activity of these novel TLR4Ls by in vitro stimulation of PBMCs or splenocytes from several species and subsequent analysis of those cells by multiplex bioluminescent assays. We used such multiplex kits that use monoclonal antibodies for the detection of secreted cytokines for those species for which these kits were available (human, mouse, rat, cynomolgus monkey). For other species (guinea pig, rabbit, ferret, cotton rat), we used a similar technology, the QuantiGene analysis kit from Affymetrix, that uses bioluminescently labeled probes to hybridize to cytokine mRNA within cell lysates and is measured using the same instrument.

**Fig 6:**
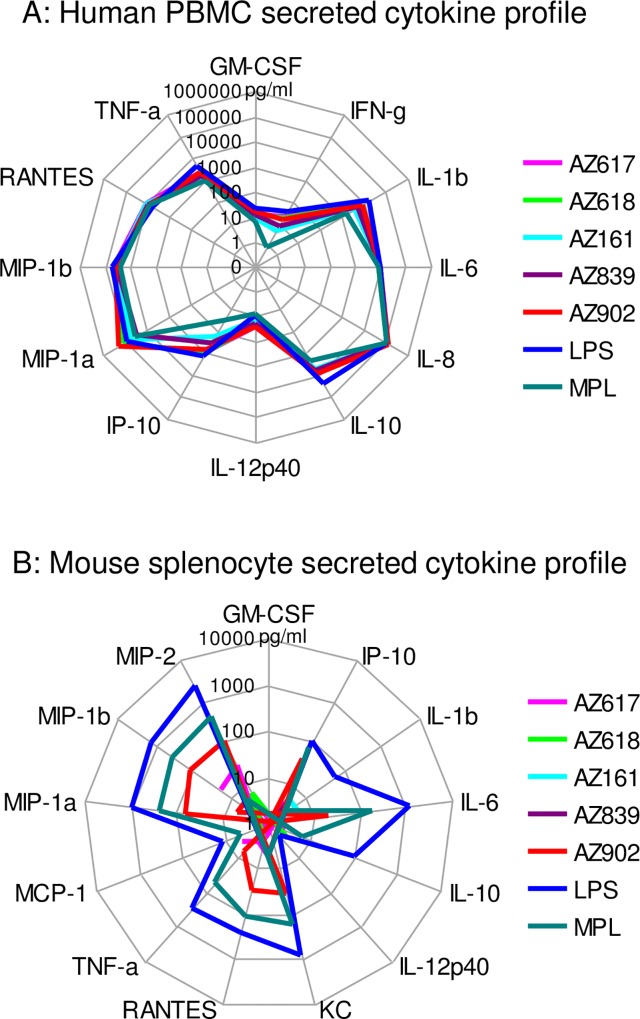
The Ugi compound-induced cytokine profile is similar to MPL and LPS in human PBMCs but not mouse splenocytes. PBMCs were isolated from three healthy volunteers, while murine splenocytes were isolated from three naïve BALB/c mice. TLR4 agonists were used at 1 and 0.2 μg/ml doses to stimulate human PBMCs and murine splenocytes for 24 hrs. SNs were analyzed via human and mouse MILLIPLEX kits. Data are expressed as the means of 3 donors/mice at the 1 μg/ml dose of TLR4 agonist and are representative of 2 studies. Raw data are presented in S3 Fig.

The specificity of Ugi compounds for the human TLR4 receptor observed with transfected HEK-293 cells was confirmed when these compounds also robustly stimulated human PBMCs to secrete several pro-inflammatory cytokines and chemokines (Fig 6A). This profile was similar to the one induced by MPL and LPS and consisted of high levels of IL-1β, IL-6, IL-8, RANTES, MIP-1α, and MIP-1β, and modest induction of TNF-α, IP-10, GM-CSF, and IL-12 p40. Ugi compounds were more potent than MPL in the induction of IL-10, IP-10, TNF-α, IL-1β, and GM-CSF, which suggests that Ugi compounds, unlike MPL, are not deficient in the activation of the MyD88 signaling pathway, which leads to NFκB translocation and expression of several pro-inflammatory cytokines. On the other hand, mouse splenocytes displayed little reactivity to Ugi compounds, save for some marginal activation by AZ902, while MPL and LPS stimulation were robust (Fig 6B and S3 Fig).

**Fig 7:**
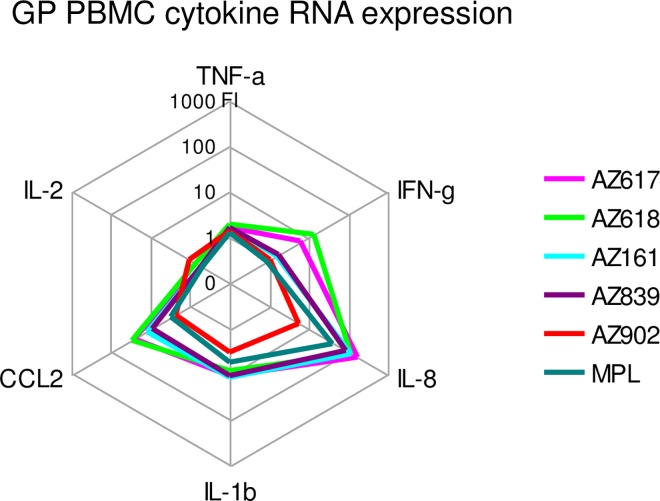
Activation of cytokine gene expression by Ugi compounds in GP PBMCs. PBMCs were isolated from three naïve GPs. TLR4 agonists were used at 1 and 0.2 μg/ml doses to stimulate GP PBMCs for 24 hrs. SNs were analyzed via GP Affymetrix QuantiGene Plex assay. Data are expressed as the means of 3 GPs at the 1 μg/ml dose of TLR4 agonist and are representative of 2 studies. Raw data are presented in S4 Fig.

Similar to the activity observed with GPTLR4-HEK293 cells (Fig 5), we confirmed that GP PBMCs also responded to Ugi compounds as measured using QuantiGene analysis of cytokine mRNAs (Fig 7 and S4 Fig). These novel TLR4 agonists induced a profile of gene expression including IFN-γ, IL-8, IL-1β, and CCL2 but little or no TNF-α and IL-2. This profile was similar to that induced by MPL. Additionally, some differences in potency between the Ugi compounds became evident in GP PBMCs, in that the highest activity is observed for AZ618 and AZ617, while AZ902 exhibited lowest activity. These results matched well with the GP HEK293 transfectant results, which also indicated AZ617 as having high levels of potency and AZ902 lower (Fig 5).

**Fig 8:**
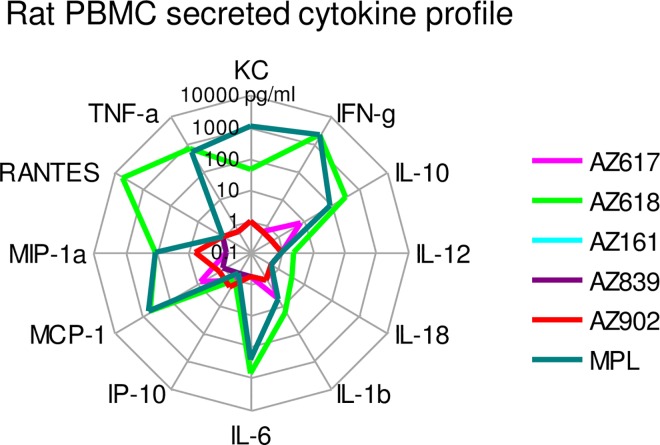
Secreted cytokine profile of rat PBMCs stimulated by AZ618. PBMCs were isolated from three naïve Sprague-Dawley rats. TLR4 agonists were used at 1 and 0.2 μg/ml doses to stimulate human PBMCs and murine splenocytes for 24 hrs. SNs were analyzed via rat MILLIPLEX kits. Data are expressed as the means of 3 rats at the 1 μg/ml dose of TLR4 agonist and are representative of 2 studies. Raw data are presented in S5 Fig.

We also tested the panel of Ugi compounds for activity on rat primary cells, by stimulating naïve rat PBMCs for 24 h and analyzing supernatants for cytokine secretion via multiplex bioluminescent analysis. Unlike guinea pigs, only AZ618 induced a response in rat PBMCs, which was comparable to that induced by MPL (Fig 8 and S5 Fig). Both MPL and AZ618 induced the production of TNF-α, IFN-γ, IL-10, IL-1β, MCP-1, and MIP-1α, with an additional contribution of RANTES to that profile by AZ618 and KC by MPL. These results suggested that the rat model demonstrates more selectivity for Ugi compound activity than human or even GP, and that the compound that was found to be active, AZ618, exhibited an activity profile that was similar to MPL in quality and quantity. Additionally, the AZ618 compound found active in rat was also one of the compounds that was optimal in GPs, identifying this compound as the best choice for in vivo development in animal models.

**Fig 9:**
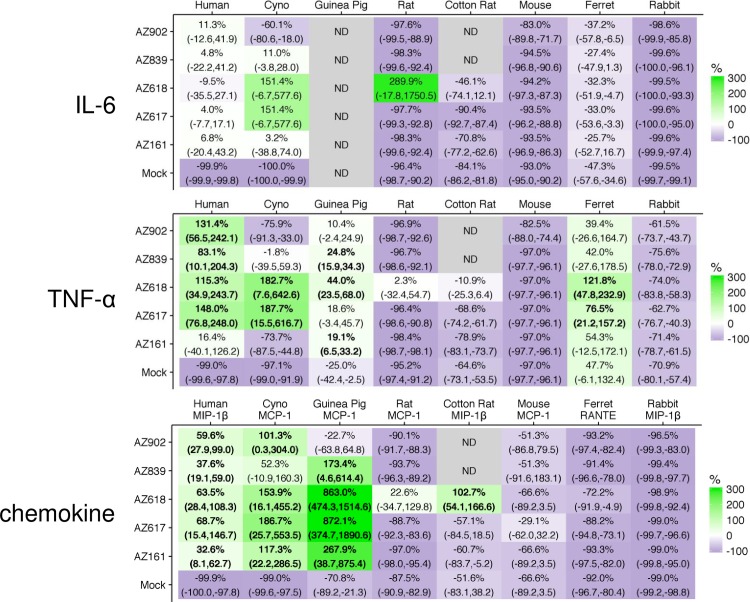
Species-specific activation of IL-6, TNF-α, and chemokine expression in primary cells. PBMCs were isolated from peripheral blood from human healthy volunteers, cynomolgus macaques, Hartley guinea pigs, ferrets, New Zealand white rabbits, Sprague-Dawley rats, and cotton rats, and splenocytes were isolated from BALB/c mice, *n* = 3 for each species, except *n* = 2 for cotton rats. TLR4 agonists were used at 1 μg/ml to stimulate primary cells of all species for 24 hrs. For human, cyno, mouse, and rat, SNs were analyzed via human, NHP, mouse, and rat MILLIPLEX kits. For guinea pig, ferret, rabbit, and cotton rat, cell lysates were analyzed via Affymetrix QuantiGene Plex kits. No one chemokine analyte was available in all 8 species-specific assays, so data were collected for any one of three different chemokines, MCP-1, MIP-1β, and RANTES. Data are expressed as mean compound % difference from the mean MPL value for that species and cytokine with associated 90% confidence interval (CI) listed below. A compound is considered significantly higher than MPL if the lower bound of the CI is higher than 0% (p value < 0.05); such values are in bold. Increasing shades of green and purple indicate increasingly higher and lower respective values than values achieved by MPL stimulation. Raw data are presented in S6 Fig.

Several other species were also tested for reactivity to Ugi compounds by Luminex-based technology with Bioplex or QuantiGene kits, which detect secreted cytokine protein and expressed cytokine mRNA, respectively. Species-specific reagents for IL-6 and TNF-α analytes were available in all species and a chemokine analyte (either MIP-1β, MCP-1, or RANTES) was selected as well (Fig 9 and S6 Fig). In addition to PBMCs from humans and guinea pigs, the cynomolgus macaque was the other species that responded broadly to the panel of Ugi molecules with a potency profile similar to human for most compounds. On the other hand, mouse splenocytes, rabbit PBMCs, and ferret PBMCs generally exhibited poor activity to the entire panel of Ugi compounds, although they did respond to MPL. Interestingly, cotton rat PBMCs demonstrated the same activity pattern as rat PBMCs: AZ618 alone of the Ugi compound panel was able to activate primary cells from those two species.

### Ugi compounds are active on mouse cells expressing hTLR4/MD2

**Fig 10:**
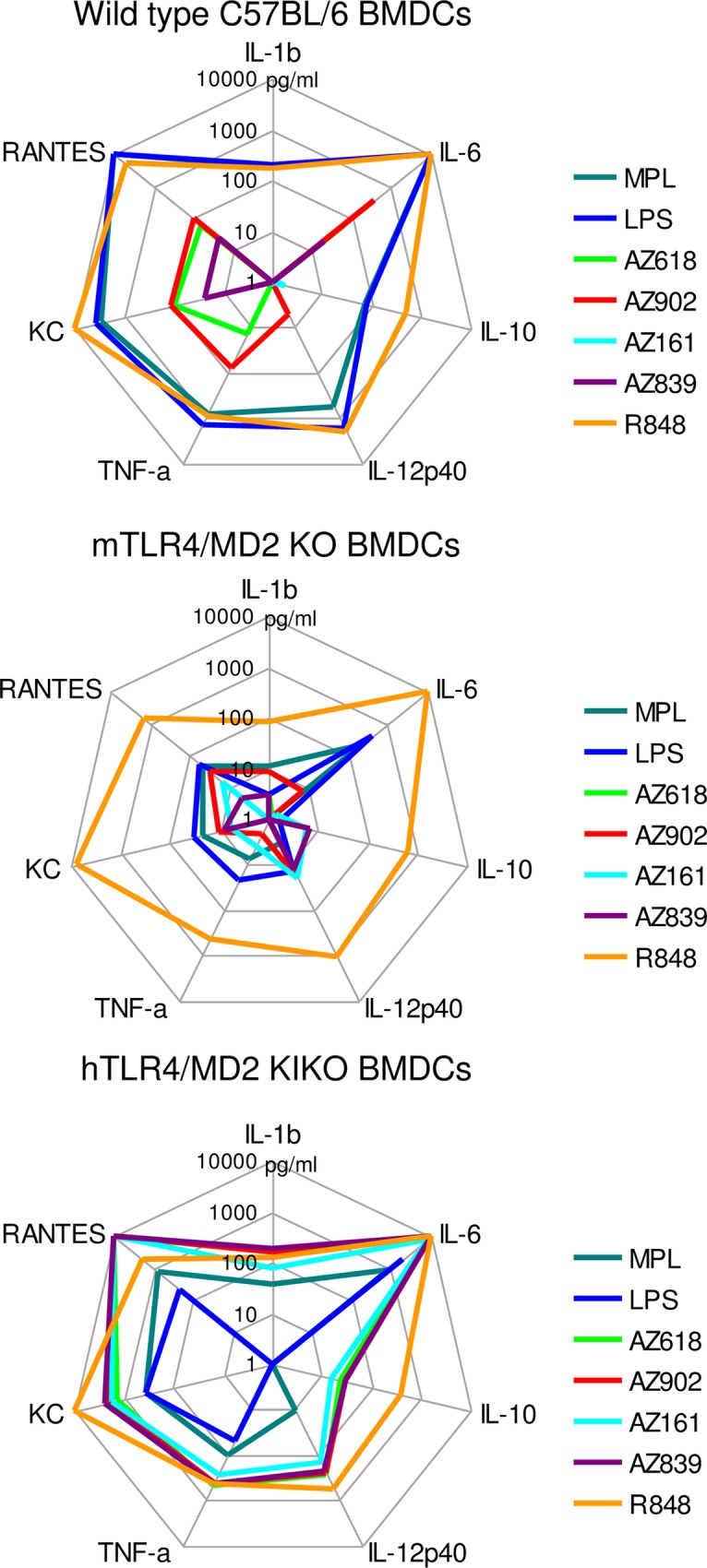
Ugi compounds activate mouse BMDCs that express human TLR4/MD2. Bone marrow cells were isolated from femur/tibia bones of w.t. C57BL/6, TLR4/MD2 KO, and hTLR4/MD2 KIKO mice and cultured with GM-CSF. BMDCs were harvested at day 9, washed, counted, and stimulated in vitro for 24 h with 1 μg/ml TLR4 agonists and 2 μM R848. SNs were analyzed via mouse MILLIPLEX kits. Data are expressed as the means of 2 mice and are representative of 2 studies.

**Fig 11:**
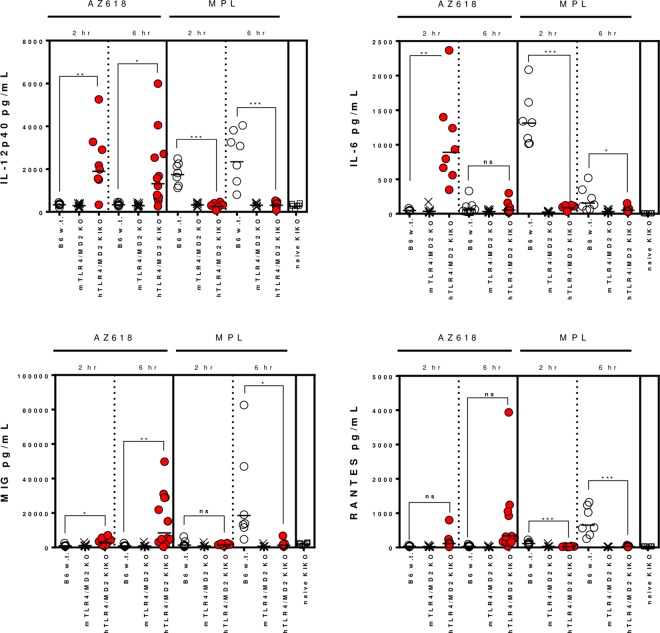
AZ618 demonstrates in vivo activity in hTLR4/MD2 KIKO mice. C57BL/6 mice (6–12 per group) were immunized IP with 50 μg MPL or AZ618 and then were bled at 2 h via retro-orbital route and at 6 h via cardiac puncture. Serum was analyzed by multiplex Millipore kits for cytokine content. Presented are individual results with a bar representing the group geometric mean with SD. Open circles represent wild-type C57BL/6 mice; X’s represent animals deficient for TLR4 and MD2; red circles represent TLR4/MD2 -/- mice transgenic for human TLR4/MD2. Statistical comparisons are between C57BL/6 w.t. and hTLR4/MD2 KIKO groups. Statistical test was one-way ANOVA performed with family-wise error rate to adjust multiple comparisons; ns, p > 0.05; *, p < 0.05; **, p < 0.01; ***, p < 0.001.

The low responsiveness of mTLR4-HEK293 transfectants and primary mouse splenocytes to Ugi compounds precludes wild-type mice as a relevant in vivo model for investigation of the immunomodulatory activity of these compounds. However, since the hTLR4 signaling complex responds with great sensitivity to triggering by Ugi compounds, we determined whether primary cells from mice expressing the human version of the TLR4/MD2 complex would respond to these compounds as equivalently as they respond to MPL. Bone marrow was acquired from transgenic mice that had been manipulated to express human TLR4 and human MD2 in the absence of murine TLR4 and MD2 [15]. BMDCs were derived from the bone marrow of wild-type mice C57BL/6, mTLR4/MD2 knockout mice, and hTLR4/MD2 “knock-in knock-out” (KIKO) mice, and stimulated with MPL, LPS, a panel of Ugi compounds, and R848, a potent TLR7 agonist (Fig 10). C57BL/6 w.t. BMDCs exhibited a strong response to MPL, LPS, and R848 with an induction profile of IL-1β, IL-6, IL-10, IL-12 p40, TNF-α, RANTES, and KC, while all Ugi compounds induced low or no responses in these cells, similar to results we observed with mouse splenocytes (Fig 6B). BMDCs derived from the mTLR4/MD2 knockout mice lost responsiveness to MPL and LPS as well but retained it to R848, as expected (Fig 10). In contrast, BMDCs from the humanized TLR4/MD2 mice demonstrated very good responsiveness to the Ugi compounds, inducing a profile of cytokine induction that was very similar to that induced by MPL and LPS on w.t. BMDCs, both in magnitude and in quality. Conversely, although MPL and LPS also retained activity on the hTLR4/MD2 KIKO BMDCs, their potency was reduced, in parallel to the lower potency these compounds exhibited on hTLR4-HEKs (Fig 4A). In vivo activity of AZ618 was also confirmed in hTLR4/MD2 KIKO mice by analysis of serum cytokines 6 hours after intramuscular administration. Fig 11 demonstrates that, as expected, MPL but not AZ618 induced IL-12 p40 and IL-6 release into serum by 2 h and IL-12 p40, MIG, and RANTES by 6 h in wild-type C57BL/6 mice. However, AZ618 induced a very similar pattern of cytokines in the hTLR4/MD2 KIKO mice, suggesting the activity profile in these mice agrees with the activity pattern of MPL in w.t. mice. MPL was much less potent in the humanized TLR4 mice, although LPS is quite active in these mice [15]. Superiority in potency of AZ618 on human TLR4/MD2 compared to MPL and LPS is also demonstrated in Figs 6A and 10. These results suggest that mice transgenic for hTLR4/MD2 can serve as an appropriate in vivo model for investigation of these compounds and may represent an environment of TLR4 sensitivity more relevant to a clinical response.

### Sequence analysis and structural modeling suggest the binding mode of the Ugi compounds that is required for the receptor activation

Because the combinatorial screens revealed that the responder/non-responder phenotype was defined primarily by the MD2 species origin rather than TLR4, we concentrated the bioinformatics analysis on the MD2 protein. The multiple sequence alignment (MSA) was built using MD2 amino acid sequences from seven species: Mus musculus, Mustela putorius furo, Oryctolagus cuniculus, Rattus norvegicus, Macaca fascicularis, Homo sapiens, and Cavia porcellus. Among those, the latter three species are responders to Ugi compounds. No MD2 sequences were found in public databases for the representatives of the genus Sigmodon, of which Cotton rat is a member. The MSA revealed several positions where the set of amino acid identities present in the responder species was non-overlapping with the identities present in the non-responder species. In order to further analyze the possible significance of these phenotype-separating sequence variations, we mapped them onto the structures of the available TLR4/MD2/LPS complexes from both human and mouse as found in the Protein Data Bank (PDB) (specific PDB IDs are reported in the Methods). Sequence positions 22, 31, 42, 49, 53, 156 and 159 were located on the surface of MD2 away from the interfaces with other subunits of the biological assembly that represents the active signaling state of the TLR4 (a homodimer of TLR4/MD2 hetero-complexes). Those residues are also distal to the binding pocket where the native LPS ligand binds. All other ligands that either activate or inhibit TLR4/MD2 signaling and have solved binding locations are found to bind in the pocket occupied by the LPS ligand [27], [24], [28], [22], [23]. Therefore, our assumption throughout the following analysis is that Ugi compounds bind in that pocket as well. Under that assumption, the positions listed above are not likely to be related to the observed phenotype evoked by the Ugi compounds.

**Fig 12:**
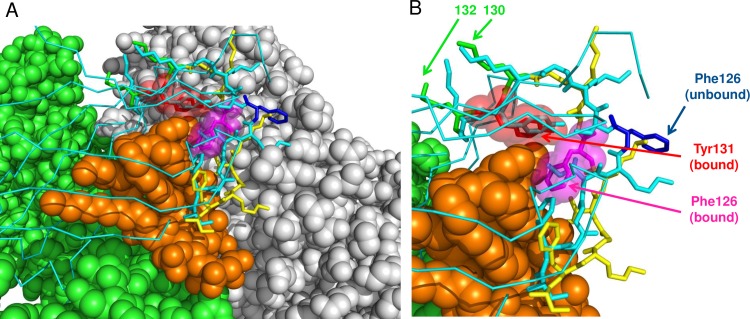
Change of conformation in a flexible loop region between residues 120 and 129 of human MD-2 upon binding to LPS ligand and dimerization with a second TLR4/MD-2 unit. Bound and unbound PDB structures were superimposed. The loop region is shown as stick representation (cyan–unbound; yellow–bound), with key residues colored separately (blue–unbound Phe126; magenta–bound Phe126; red–bound Tyr131; green–bound 130 and 132 residues). Bound Phe126 and Tyr131 are additionally rendered with semi-transparent spheres. The rest of MD-2 is shown as backbone trace and only for the unbound structure (cyan). Solid spheres show LPS ligand (orange), TLR4 of the first unit (chain A, green) and TLR4 of the second unit (grey). A) zoom out view with the entire LPS ligand; B) zoom in view with the second TLR4 unit hidden in order to provide better visibility for the conformation of the flexible loop of MD-2.

Position 86 is at the edge of the dimerization interface (that is, in contact with chain B in our reference PDB structures), but is outside of the binding pocket. Positions 130 and 132 require more attention. Residue 130 is conserved in the responder species (Lys), and variable in the non-responders (2 His, 1 Arg and 1 Gln), while 132 is conserved in the non-responders (Arg) and variable in the responders (2 Lys and 1 Tyr). The side chains of both 130 and 132 residues are on the outer surface of the MD2, outside of the binding pocket or any interfaces (Fig 12). However, the residue between these two positions, 131, is a universally conserved Tyr (except for Rattus norvegicus where it is a His), and its side chain protrudes inside the binding pocket in the corner that is close to the dimerization interface. We also note that rat, where this residue is not conserved, is only a partial non-responder (activated by one Ugi compound out of five). Thus, the positions 130 and 132 may be those that define the Ugi-related phenotype. Their influence must be carried out indirectly through the backbone by affecting the conformation of the Tyr131, and the latter could be critical for the binding of the Ugi compounds. We note that the conformations of these residues observed in bound and unbound human and mouse PDB structures are very close–they can be aligned within less than 0.3 Å RMSD. However, side-chain of Tyr131 is in close proximity with the side chain of Phe126 in the known ligand-bound structures. The loop region containing Phe126 and stretching from residue 120 to residue 129 undergoes a backbone conformational change upon binding with either one of LPS-like or Neoseptin-3 ligands [24]. Upon binding, the side chain of Phe126 flips and is directed inside the binding pocket (Fig 12B). Thus, even subtle changes in the conformation or flexibility of Tyr131 may potentially have a profound influence on the dimerization and activation of the TLR4/MD2 complex if that residue is involved in the binding between Phe126 and the ligand. The identity of residue 132 can also influence directly the conformation of the flexible loop region 120–129. The multiple sequence alignment of MD2 protein in responder and non-responder species are provided in a supplemental archive S1 File.

**Fig 13:**
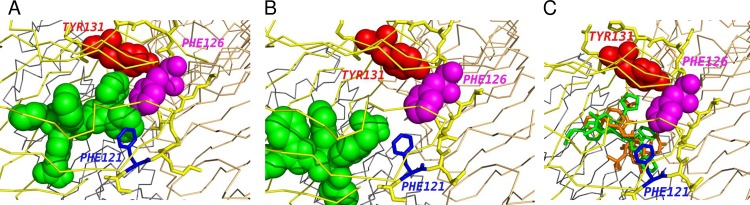
Representative docking models. Ugi ligand is green, other key residues—as labeled on the Fig The rest of MD-2 is yellow backbone trace with the flexible loop region shown as sticks. A) Docking mode where Ugi compound is predicted to interact with MD-2 residues Tyr131 and Phe126 (best-scored model for AZ617 docked into human receptor). B) Alternative docking mode where Ugi compound is deep inside the binding pocket away from Tyr131 and dimerization interface (best-scored model for AZ617 and the mouse receptor). C) Comparison of the docked Ugi compound and crystallographically solved Neoseptin-3 ligand in complex with the mouse receptor (model 7 from AZ161-mouse docking). MD-2 coordinates from both complexes were superimposed. AZ161—green sticks, Neoseptin-3—orange sticks.

In order to have independent computational validation of our hypothesis that the action of Ugi compounds is defined by sequence variations in MD2 positions 130 and 132, we performed a series of docking simulations. We selected a large docking search area that contained the entire LPS binding pocket, and repeated the same docking protocol for each of the five Ugi compounds with human (known responder to Ugi) and mouse (known non-responder) receptors. The search was completely unbiased within the confines of the docking area. For the receptor structure, we used:—ligand-free (non-dimerized) PDB entries;—dimerized entries solved with the LPS ligand (after removing the LPS);—and the same dimerized entries with the second TLR4/MD2 subunit (and the LPS) removed. Docking with both ligand-bound and ligand-free PDB structures of the TLR4/MD2 receptor was done to account for the binding-induced change of the Phe126 loop region. Each of the simulations predicted two distinct clusters for the locations of the Ugi compound. Those clusters are very close for all combinations of receptors and ligands that we tried (Fig 13A and 13B show representative models). In the first cluster (Fig 13A), the ligand is in contact with the phenyl rings Tyr131, Phe126, as well as Phe121. Phe126 moves into this position only upon binding and dimerization in the solved complexes of LPS and its agonists (Fig 12). The particular model in Fig 13A shows a remarkable interlocked arrangement of five phenyl rings contributed by AZ617 and MD2. The pairwise orientations of the rings are consistent with those frequently observed in PDB protein-ligand structures [29]. In the second cluster (Fig 13B), the ligand is located deeply in the opposite corner of the hydrophobic pocket on MD2, away from Tyr131, Phe126, and the dimerization interface. The median difference of the docking scores between these two distinct binding modes within the same simulation is 0.2 Kcal/mol, and it is always less than 0.8 Kcal/mol. The known standard deviation of the AutoDock Vina forcefield is 2.5 Kcal/mol. Thus, within the accuracy available to us under the docking simulation, the two alternative binding modes are energetically equal. Note that the Smina/Vina/Autodock forcefields were by design calibrated to rank different binding modes either within the same receptor-ligand pair, or between different ligands with the same receptor. The forcefield is not designed to rank binding of different receptors with the same ligand. This is why we focus on the positional preference of the ligand within a large binding pocket in our interpretation of the docking results. The inputs and outputs of the docking simulations are provided in a supplemental archive S1 File.

A recent work [24] has reported the synthetic peptidomimetic ligand Neoseptin-3 causes a dimerization of TLR4/MD2 and activation of TLR4 signaling in mice, with a ligand-binding mode distinct from that of the native LPS molecule. Neoseptin-3 itself binds MD2 as a dimer. When we superimposed the available complex of TLR4/MD2/Neoseptin-3 with the receptor that we used in our simulations, we saw a substantial overlap between our predicted position of the Ugi compounds and the Neoseptin-3 (Fig 13C). In particular, Tyr131 and Phe126 form contacts with the ligand in both cases, suggesting that interactions between Ugi molecules and MD2 residues may be similar to what is observed with Neoseptin-3.

## Discussion

This paper describes the discovery of a new class of small molecule compounds generated through the Ugi synthesis reaction that triggers the human TLR4 signaling complex on HEK293 transfectant cells and on human PBMCs. Responsiveness of the mouse TLR4 complex, however, was low or absent on transfectants and murine splenocytes. A species-specific pattern was observed in which human, cynomolgus monkey, and guinea pig cells responded robustly to most or all Ugi compounds, while mouse, ferret, and rabbit cells were very unresponsive to the entire compound panel. The responsiveness of rat and cotton rat cells was specific for the AZ618 compound only. Through the use of cross-species HEK293 transfectants, the TLR4 accessory molecule MD2 was determined to be the critical component that conferred triggering activity for Ugi compounds. Moreover, mouse cells transgenic for human TLR4/MD2 on a background of mouse TLR4/MD2 deficiency responded strongly in vitro and in vivo to Ugi compound stimulation, indicating that mouse cells can be suitable as a substrate given the expression of a species-preferred TLR4 complex.

The prototypical TLR4 agonist is represented by LPS, a cell wall component found in Gram-negative bacteria, typically encountered during an infection in soluble form. LPS is shed from the bacterial cell wall and bound by the soluble acute phase protein LBP (LPS-binding protein), a chaperone-like molecule that shuttles the LPS molecule from LPS aggregates to the surface of a macrophage or dendritic cell. There, the accessory molecule CD14 transfers LPS from LBP to a complex of TLR4 and MD2, associated with the extracellular domain of TLR4. LPS binding induces the heterodimerization of TLR4/MD2 [8] and signaling is initiated with activation of the TIRAP-MyD88 and TRAM-TRIF pathways [2]. Derivatives of LPS, such as the lipid A moieties MPL or GLA, appear to retain the same general mechanism of interaction with these elements in order to achieve TLR4 activation. However, Ugi compounds appear to bypass the requirement for transfer by CD14, since their activation of TLR4-HEK transfectants is not significantly impaired by the absence of co-expression of CD14, unlike what is observed for LPS, which requires the presence of CD14 for full activity (Fig 5). In addition, although TLR4-dependent, the species-specific human MD2 appears to be critical for Ugi compound activity such that hMD2, when associated with either hTLR4 or mTLR4, can mediate optimal activity, while mMD2 is less effective (Fig 4C). On the other hand, LPS and MPL display a preference for signaling based on the species of TLR4 (mouse), which is superior to hTLR4 regardless of the species of MD2 associated. Thus, the Ugi compounds demonstrate that some variation can exist in the traditional operation of the LPS-TLR4 activation pathway while still achieving robust signaling.

Although both MPL and Ugi compounds trigger TLR4 signaling, they are structurally dissimilar. MPL is an amphiphilic lipid with 6 hydrophobic acyl chains while Ugi compounds are much smaller molecules with approximately one-third of the mass of MPL with aromatic rings and structural similarities to amino acids. This ligand diversity is consistent with the often reported promiscuousness of the TLR4 receptor, which is known to count among its ligands such structurally diverse compounds as host-derived heat shock protein 60 [30], products of fibronectin degradation [31], the tubulin-targeting drug Paclitaxel [32] derived from yew bark, the ubiquitous large molecule glycosaminoglycan hyaluronic acid [33], and some viral antigens such as the F antigen from the respiratory syncytial virus (RSV) and the mouse mammary tumor virus (MMTV) envelope protein [34], [4]. The dissimilarity in structure between traditional MPL-like lipid molecules and Ugi compounds resembles a similar difference observed among TLR7 agonists. Natural TLR7Ls are sections of guanidine- and uridine-rich single-stranded viral RNA, degraded in the endosomal compartment, but it has been observed for years that small synthetic molecules like imidazoquinolines [35] and 8-oxoadenine derivatives [36] can be potent triggers for TLR7 despite their much smaller size and mass (250–350 kDa compared to <1 kDa).

A distinct pattern of species preference was observed for Ugi compounds in that activation of human and cynomolgus monkey primary cells was robust while TLR4 triggering of mouse, ferret, and rabbit primary cells proved much weaker. Species preference has been reported for other TLR4 agonists. We observed that LPS induced higher activity on mTLR4-expressing than hTLR4-expressing HEK293 cells, regardless of the species of co-expressed MD2 (Fig 4C). TLR4 recognition of differentially acylated forms of LPS has been traced to a hypervariable 82 amino acid domain within the extracellular region of TLR4 that may have undergone differential natural selection pressures between species due to the cohort of Gram-negative bacterial challenges experienced [37]. In addition, hypoacylated precursor forms of LPS, such as tetraacylated lipid IVa, are known to have activity on mouse TLR4 and not human TLR4 [22]. This has been linked to different surface charges between human and mouse MD2, several residues of which have been found poorly conserved between the two species [38]. The negatively-charged Glu122 residue of mMD2, located in the cavity entrance, interacts with the charged phosphate group of lipid IVa but when converted to the positively charged human residue Lys122, responsiveness is lost [39]. Furthermore, the human and mouse versions of MD2 differ in the structural dimensions of their hydrophobic pockets and the surface charges of their dimerization interface with TLR4, and substitution of mouse for human residues in this area also results in an inability to respond to lipid IVa [40]. Such observations suggest that the hydrophobic potentials and charge distributions of MD2 interfaces involved in dimerization are critical for ligand recognition by the TLR4 complex and successful activation of the signaling pathway. It is possible that the Ugi compounds may interact with MD2 by a mechanism similar to that of lipid IVa, in that one species of MD2 (in this case human) interacts successfully with the Ugi compound to activate TLR4 signaling while the other species (mouse) does not. Previously, examples of this kind such as lipid IVa and taxol have demonstrated preferential activity for mouse MD2 at the expense of human MD2 but our data with Ugi compounds reveals that TLR4Ls that favor the reverse phenotype also exist.

Mapping the variable positions in the multiple sequence alignment of MD2 across responder and non-responder groups of species onto the available TLR4/MD2 crystallographic structures suggested the critical role of sequence variations at positions 130 and 132. Observed group-wise differences in the identity of these residues likely affect the behavior of the conserved residue 131 between them, which is a Tyr in all responders and non-responders except the rat. The side chain of that residue is located inside the hydrophobic binding pocket of MD2 and is in contact with Phe126. The latter is located in a loop that undergoes a large conformational change upon binding of different types of ligands known to cause further dimerization of the TLR4/MD2 complex and activation of the TLR4 signaling pathway.

Unconstrained docking of Ugi compounds to a large area enclosing the hydrophobic pocket of MD2 identified two alternative preferential positions for the ligand. One was deeper in the hydrophobic pocket and away from both Tyr131 and the dimerization interface. Another position was in direct contact with Tyr131 in docking results for both bound and unbound PDB receptor structures in both human and mouse. Tyr131 was also in contact with Phe126 when the bound receptor structures were used for docking simulation in both species. The binding energy estimated by the docking algorithm was indistinguishable between these two alternative binding modes within the accuracy of the force field. The Tyr131-related binding mode exhibited pronounced similarity with the TLR4 peptomimetic ligand Neoseptin-3, as observed in its complex with TLR4/MD2 available in PDB.

Therefore, the computational analysis provides evidence for the hypothesis that consistent changes at residue positions 130 and 132 between responder and non-responder species affect the interaction of the Ugi compounds with the conserved residues 131 and 126. The predicted three-way contacts between any of the Ugi ligands and residues 131 and 126 may be critical for causing the large conformational change in the loop containing residue 126, the change that is observed upon binding of other known agonists. This mechanism could explain how the difference in sequences of MD2 translates into the difference in activation that we observed between species. The available docking forcefield is not sufficiently sensitive for evaluating the change in binding energies between responder and non-responder species. It is not possible to estimate if the Ugi compounds bind the receptor in the non-responder species in the alternative location shown on Fig 13B or do not bind at all. Considering the yet unsolved methodological challenges in computational docking wherever a substantial backbone conformational change of the receptor is involved, the definitive proof for the binding mechanism illustrated in Fig 13A can only be obtained by means of experimental techniques like directed mutagenesis of residues 130 and 132 and protein crystallography of the receptor-Ugi complexes.

Although PDB complexes for both LPS and Neoseptin-3 [24] demonstrate the ligand to be in contact with the convex face of the second TLR4 subunit (biounit chain B) in the dimerized complex, we observed such contacts only in a minority of Tyr131-bound Ugi orientations predicted by our docking simulations. This was not surprising considering that Ugi was a smaller compound. It is not clear if such contact is necessary for homo-dimerization of TLR4/MD2 hetero-complex, or if the Phe126 loop change can be sufficient by itself. It is also possible that Ugi compounds themselves bind as dimers similarly to what is observed for Neoseptin-3.

Classical TLR4Ls like LPS activate two signaling cascades commonly referred to as the MyD88-mediated and TRIF-mediated pathways. Engagement of TLR4 at the surface recruits MyD88 and leads to a signaling cascade with successive activation of TRAF6, TAK1, IKK, and ending with the nuclear translocation of activated NFkB, which promotes the transcription of a host of inflammatory mediators including TNF-α, IL-6, and IL-1β [1], [41]. On the other hand, TLR4 triggering from an endosomal vesicle results in recruitment of TRIF, activation of TBK1 and then of IRF3 [1], [41], a transcription factor known to be essential for the expression of IFN-β, IP-10, and G-CSF [3]. Many of the gene products from activation of the NFκB pathway are known to be directly responsible for high inflammatory responses that can lead to toxicity. However, MPL, a low-toxicity derivative of LPS, has been engineered to subvert higher activation of NFκB-mediated signaling while maintaining optimal induction of the TRIF pathway [3]. We observed that Ugi compounds induced expression of both sets of cytokines in human PBMCs (Fig 6), including higher production of MyD88-dependent IFN-γ, TNF-α, IL-1β, compared to MPL.

Other high throughput screenings have recently identified additional families of small molecule TLR4 agonists, structurally unrelated to Ugi compounds, known as 4-aminoquinazolines [42] and pyrimido[5,4-b]indoles [43]. These compounds have neutral charge with IC50 values on hTLR4-HEK transfectants in the range 1–10 μM [43] and thus approximately 2 orders of magnitude less potent than the Ugi compounds (Fig 3). In regards to in vitro activity, the profile of both Ugi compounds and 4-aminoquinazolines on TLR4-HEK transfectants and primary cells similarly demonstrates a strong signaling preference for human TLR4/MD2 over the mouse equivalent, dispensability of CD14 for signaling, and rescue of signal by transfection of hTLR4/MD2 into mouse cells [42], although pyrimido[5,4-b]indoles demonstrated a mouse over human preference [43]. Therefore, it seems possible that these three sets of compounds interact with and trigger TLR4 in similar fashion, although definitive conclusions must wait until direct comparisons can be made. However, our results extend the observations that these types of compounds are active in vivo in a TLR4-specific manner when human TLR4/MD2 is expressed. Since the mouse model is predominant in preclinical adjuvant testing, the lack of activity of these compounds in wild-type mice is problematic for further in vivo development. However, we have identified two species, guinea pig and rat, for which at least some of the Ugi compounds are active, and these species could be developed further as in vivo models for testing the immunomodulatory and adjuvant activity of Ugi compounds and, possibly, 4-aminoquinazolines and pyrimido[5,4]indoles. No pharmacokinetic (PK) or in vivo data were reported for either of these series of compounds, but it might be expected that their PK profile would be inferior compared to the acidic Ugi compounds based on the assumption that the charged group retained during Ugi synthesis has a major benefit in reducing lipophilicity (LogD) and improving metabolic clearance. It should also be noted that both series contain metabolically vulnerable groups (i.e. the ester in the 4-aminoquinazolines [44] and the sulfur in the pyrimido[5,4-b]indoles) and, as these groups are necessary for potency, further substantial optimization may be required before either series could be useful for in vivo studies, although this awaits confirmatory data. Additionally, before any of the 4-aminoquinazoline series compounds could be considered as potential drugs, it may be necessary to replace the key aromatic nitro group, as such compounds have a correlation with unacceptable toxicity effects including mutagenicity [45], hepatotoxicity [46] and acute toxicity [47].

Several reports have indicated that the manner of delivery of a TLR agonist within a formulation delivery vehicle can greatly enhance the adjuvant properties of the immunomodulator molecule. Most known TLR4 agonists are based on the lipid A prototype of an oligosaccharide with 4–7 lipophilic fatty acid side chains, which facilitate incorporation into liposomes or oil-based emulsions. This integral positioning of the TLR4 agonist within a particulate substrate has demonstrated enhanced adjuvant properties, notably in the case of GLA/SE, in which the synthetic purified hexa-acylated glucopyranosyl lipid A is incorporated into the squalene-based oil-in-water emulsion (SE). The aqueous insolubility of lipid A structures that encourages compatibility with lipid-based substrates also hinders their encapsulation into polymer nanoparticles, a rapidly developing area of adjuvant development that has the advantage of packaging antigens and one or more immunomodulators together to ensure simultaneous delivery to target APCs [48], [49], [50]. The superior water solubility of Ugi compounds over lipid A analogs should facilitate the loading of nanoparticle formulations with TLR4-specific payloads. Additionally, the synthesis protocol for Ugi compounds allows for a simpler generation of a wide diversity of species simply by altering the amine group as one of the starting components, while diversity generation in lipid A structures requires identifying bacterial strains that naturally or are engineered to produce an LOS species of interest, which then would need to be further refined to the lipid A analog. Finally, the dispensability of CD14 in Ugi compound activity could allow for greater impact of TLR4 adjuvant effects on TLR4^+^ target cells that generally do not express high levels of CD14, such as dendritic cells, the initiator cell-type of most adjuvant-fueled immune responses.

TLR4 agonists have the most extensive regulatory approval record of all TLR-specific compounds and several TLR4 agonists have been tested in human subjects, mostly detoxified derivatives of LPS, including MPL, E2060, Ribi, and GLA [1]. AS04 is the first described TLR agonist-based adjuvant approved by the FDA and consists of aluminum salts formulated with MPL [51]. Further investigation of the use of detoxified LPS variants either alone or in formulation with delivery vehicles continues on multiple fronts. The discovery of Ugi compounds as human-specific TLR4 agonists with superior potency to MPL reveals a new category of ligand that can be investigated for use in engineering potently human-active but safe adjuvants.

## Supporting Information

S1 Fig Ugi compounds demonstrate specificity for human TLR4. Several TLR agonists specific for TLR2 (Pam3CSK4), TLR4 (PHAD), TLR7 (R848, Gardiquimod), and TLR9 (CpG-2395) were used to stimulate hTLR2-, hTLR4-, hTLR7-, hTLR9-HEK cell lines at a dose response range of 1 μg/ml to 0.01 μg/ml. 24 h later, 20 μl supernatants were transferred to wells containing 180 μl QUANTI-Blue (InVivogen), incubated 1–2 h 37°C, and read on a VERSAmax microplate reader (Molecular Devices) at 650 nm.
(TIF)

S2 Fig Ugi compounds demonstrate requirement for MD2 expression but not CD14. (A-B) Stably-transfected hTLR4-HEK cells were supplemented or not with conditioned media (25% by volume) from hMD2-HEK cells and stimulated with Ugi compounds (10 to 0.078 nM) and LPS (1 to 1e-7 ng/ml) for 48 h. (C-D) Stably-transfected hTLR4-HEK cells were supplemented with conditioned media (25% by volume) from hMD2-HEK cells and with conditioned media from hCD14-HEK cells (serial dilutions starting at 25% by volume). Cells were stimulated with AZ606 (2.5 to 1.48e-5 nM) or LPS (250 to 0.001 ng/ml) for 48 h. Similar results were obtained with AZ842 and AZ462. (A-D) 50 μl SN was added to alkaline phosphatase yellow (pNPP) liquid substrate and absorbance measured at 405 nm over 15 min using a Versamax reader. Data are expressed as the Vmax of secreted embryonic alkaline phosphatase (SEAP) on pNPP substrate.
(TIF)

S3 Fig Secreted cytokine profile of human and mouse primary cells stimulated by Ugi compounds. Mock refers to unstimulated cells. Upper panel: Data presented in Fig 6 are expressed as mean compound % difference from the mean MPL value for that cytokine with associated 90% confidence interval (CI) listed below. A compound is considered significantly higher than MPL if the lower bound of the CI is higher than 0% (p value < 0.05); such values are in bold. Increasing shades of green and purple indicate increasingly higher and lower respective values than values achieved by MPL stimulation. Lower panel: Data presented in Fig 6 are here represented as bar graph means with SEM bars.
(TIF)

S4 Fig Secreted cytokine profile of GP PBMCs stimulated by Ugi compounds. Mock refers to unstimulated cells. Upper panel: Data presented in Fig 7 are expressed as mean compound % difference from the mean MPL value for that cytokine with associated 90% confidence interval (CI) listed below. A compound is considered significantly higher than MPL if the lower bound of the CI is higher than 0% (p value < 0.05); such values are in bold. Increasing shades of green and purple indicate increasingly higher and lower respective values than values achieved by MPL stimulation. Lower panel: Data presented in Fig 7 are here represented as bar graph means with SEM bars.
(TIF)

S5 Fig Secreted cytokine profile of rat PBMCs stimulated by Ugi compounds. Mock refers to unstimulated cells. Upper panel: Data presented in Fig 8 are expressed as mean compound % difference from the mean MPL value for that cytokine with associated 90% confidence interval (CI) listed below. A compound is considered significantly higher than MPL if the lower bound of the CI is higher than 0% (p value < 0.05). Increasing shades of green and purple indicate increasingly higher and lower respective values than values achieved by MPL stimulation. Lower panel: Data presented in Fig 8 are here represented as bar graph means with SEM bars.
(TIF)

S6 Fig Species-specific activation of IL-6, TNF-α, and chemokine expression in primary cells. Data presented in Fig 9 here represented as bar graph means with SEM bars. Mock refers to unstimulated cells.
(TIF)

S1 File Supplemental Archive. Sequence and structure files used for bioinformatics analysis and docking, provided as a ZIP archive of a directory tree. To view, uncompress onto your local file system and open the file Readme.txt at the top directory level of the archive. That file describes individual directories and files inside the archive.
(ZIP)

## Abbreviations

CR: cotton rat
GLA: glucopyranosyl lipid A
GP: guinea pig
HEK: human embryonic kidney
KIKO: knock in-knock out
LBP: LPS-binding protein
LPS: lipopolysaccharide
MD2: myeloid differentiation 2
MPL: monophosphoryl lipid A
PDB: Protein Data Bank
PHAD: phosphorylated hexaacyl disaccharide
SN: supernatant
TLR4: Toll-like receptor 4
TLR4L: TLR4 ligand
