# Supplementary figures and images for: A Novel Class of Small Molecule Agonists with Preference for Human over Mouse TLR4 Activation
Source: PLoS One. 2016 Oct 13;11(10):e0164632. doi: 10.1371/journal.pone.0164632 (PMC5063506; doi:10.1371/journal.pone.0164632)

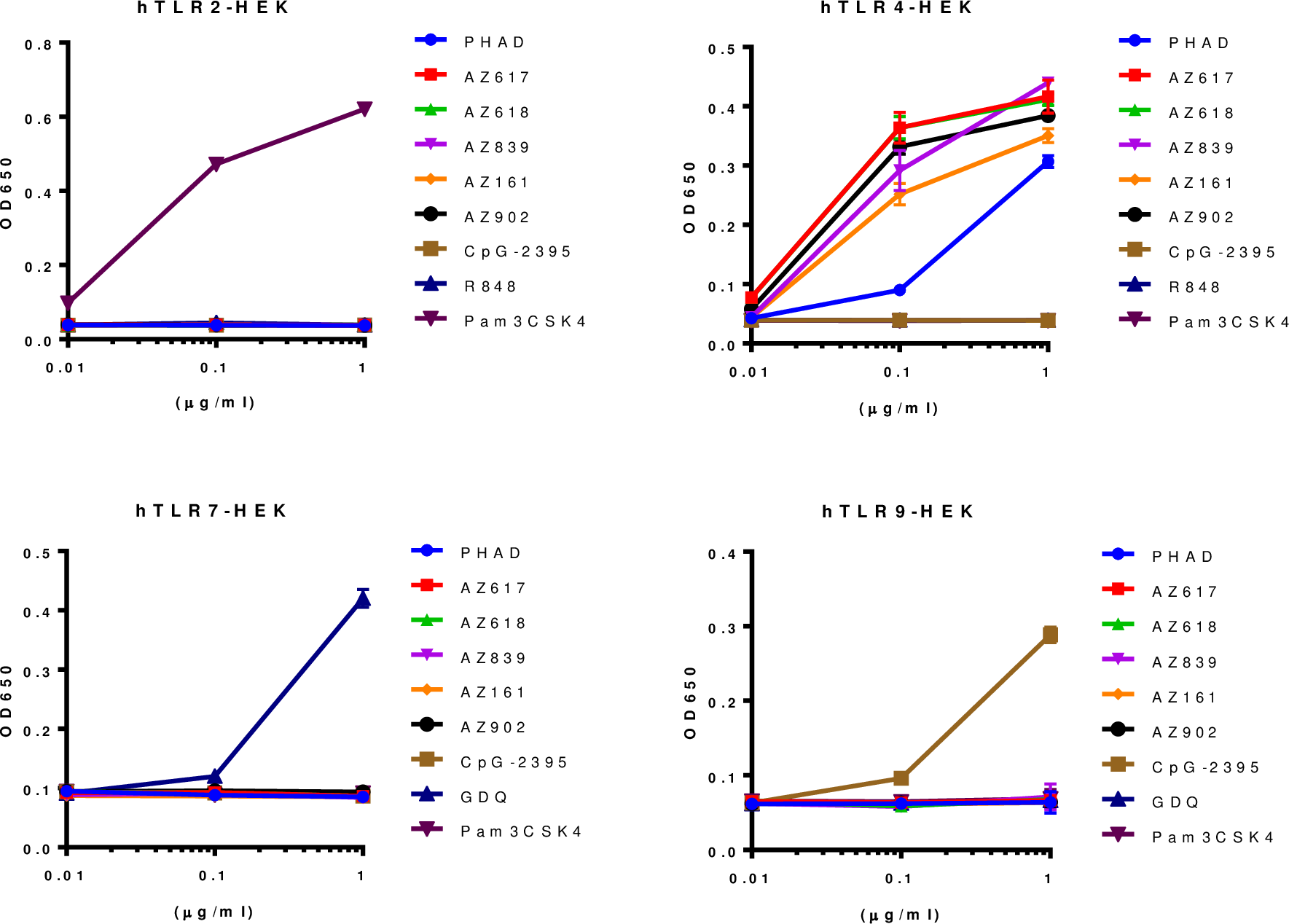

Supplement: S1 Fig — Several TLR agonists specific for TLR2 (Pam3CSK4), TLR4 (PHAD), TLR7 (R848, Gardiquimod), and TLR9 (CpG-2395) were used to stimulate hTLR2-, hTLR4-, hTLR7-, hTLR9-HEK cell lines at a dose response range of 1 μg/ml to 0.01 μg/ml. 24 h later, 20 μl supernatants were transferred to wells containing 180 μl QUANTI-Blue (InVivogen), incubated 1–2 h 37°C, and read on a VERSAmax microplate reader (Molecular Devices) at 650 nm. (TIF) [file pone.0164632.s001.tif]

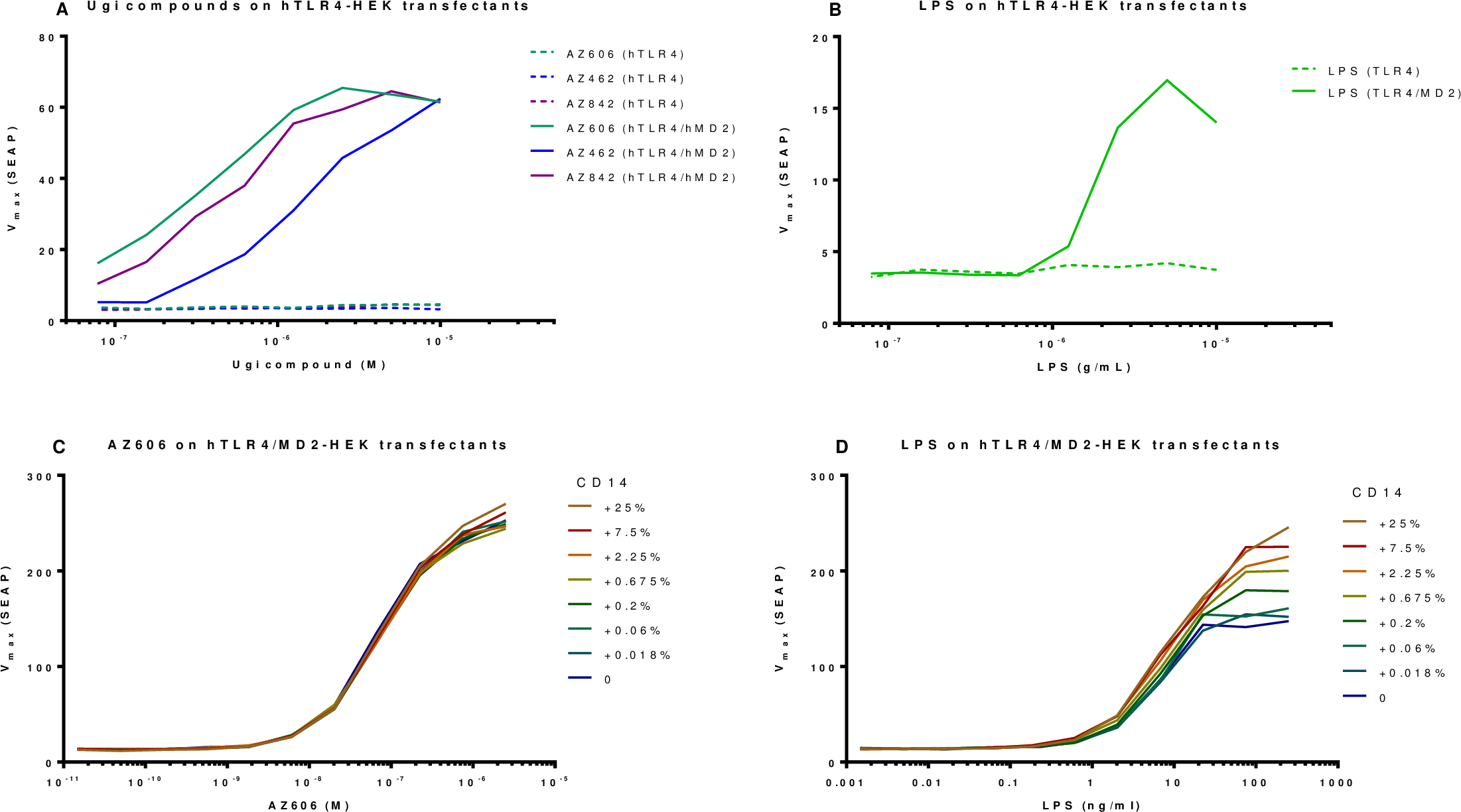

Supplement: S2 Fig — (A-B) Stably-transfected hTLR4-HEK cells were supplemented or not with conditioned media (25% by volume) from hMD2-HEK cells and stimulated with Ugi compounds (10 to 0.078 nM) and LPS (1 to 1e-7 ng/ml) for 48 h. (C-D) Stably-transfected hTLR4-HEK cells were supplemented with conditioned media (25% by volume) from hMD2-HEK cells and with conditioned media from hCD14-HEK cells (serial dilutions starting at 25% by volume). Cells were stimulated with AZ606 (2.5 to 1.48e-5 nM) or LPS (250 to 0.001 ng/ml) for 48 h. Similar results were obtained with AZ842 and AZ462. (A-D) 50 μl SN was added to alkaline phosphatase yellow (pNPP) liquid substrate and absorbance measured at 405 nm over 15 min using a Versamax reader. Data are expressed as the Vmax of secreted embryonic alkaline phosphatase (SEAP) on pNPP substrate. (TIF) [file pone.0164632.s002.tif]

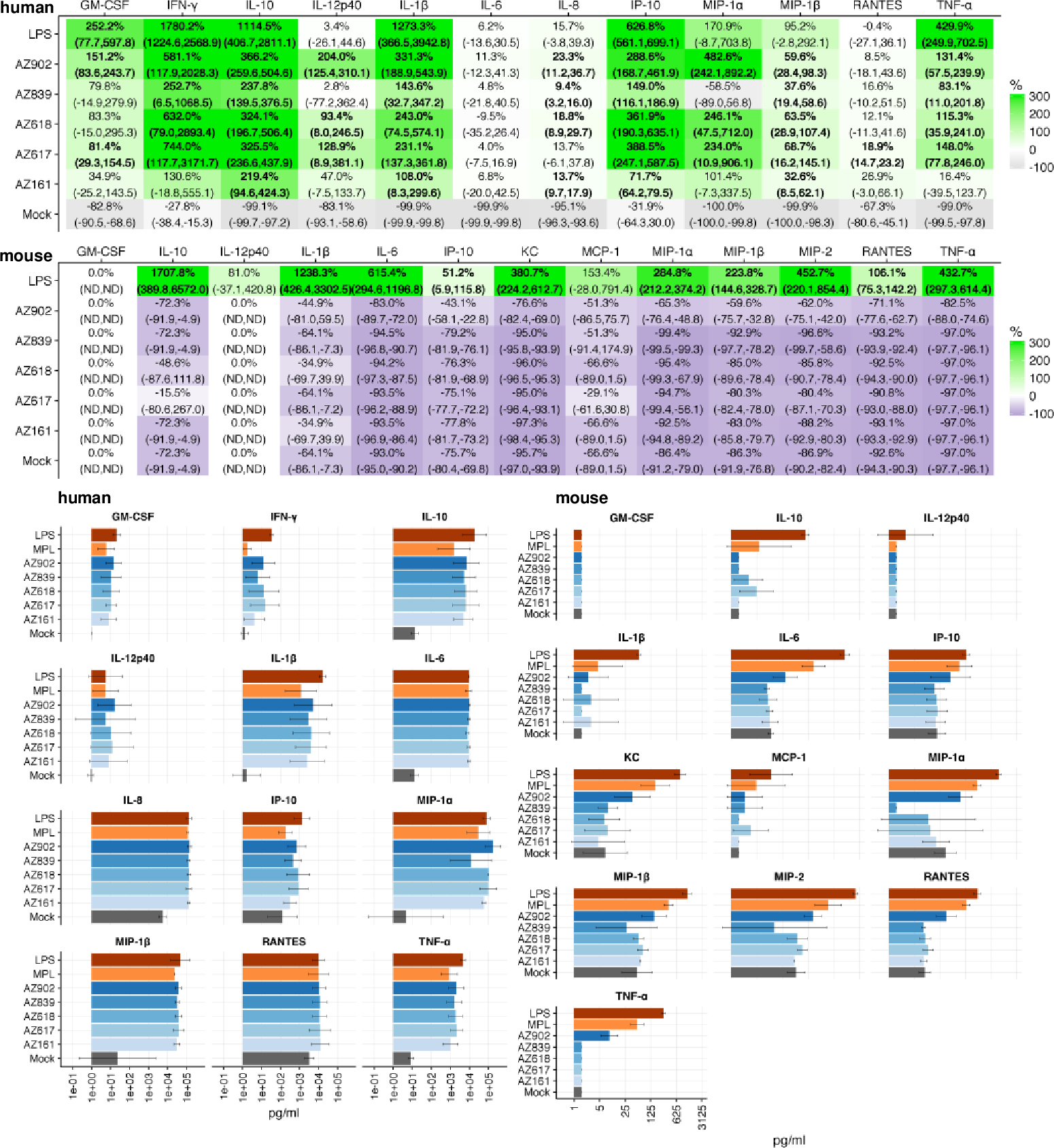

Supplement: S3 Fig — Mock refers to unstimulated cells. Upper panel: Data presented in Fig 6 are expressed as mean compound % difference from the mean MPL value for that cytokine with associated 90% confidence interval (CI) listed below. A compound is considered significantly higher than MPL if the lower bound of the CI is higher than 0% (p value < 0.05); such values are in bold. Increasing shades of green and purple indicate increasingly higher and lower respective values than values achieved by MPL stimulation. Lower panel: Data presented in Fig 6 are here represented as bar graph means with SEM bars. (TIF) [file pone.0164632.s003.tif]

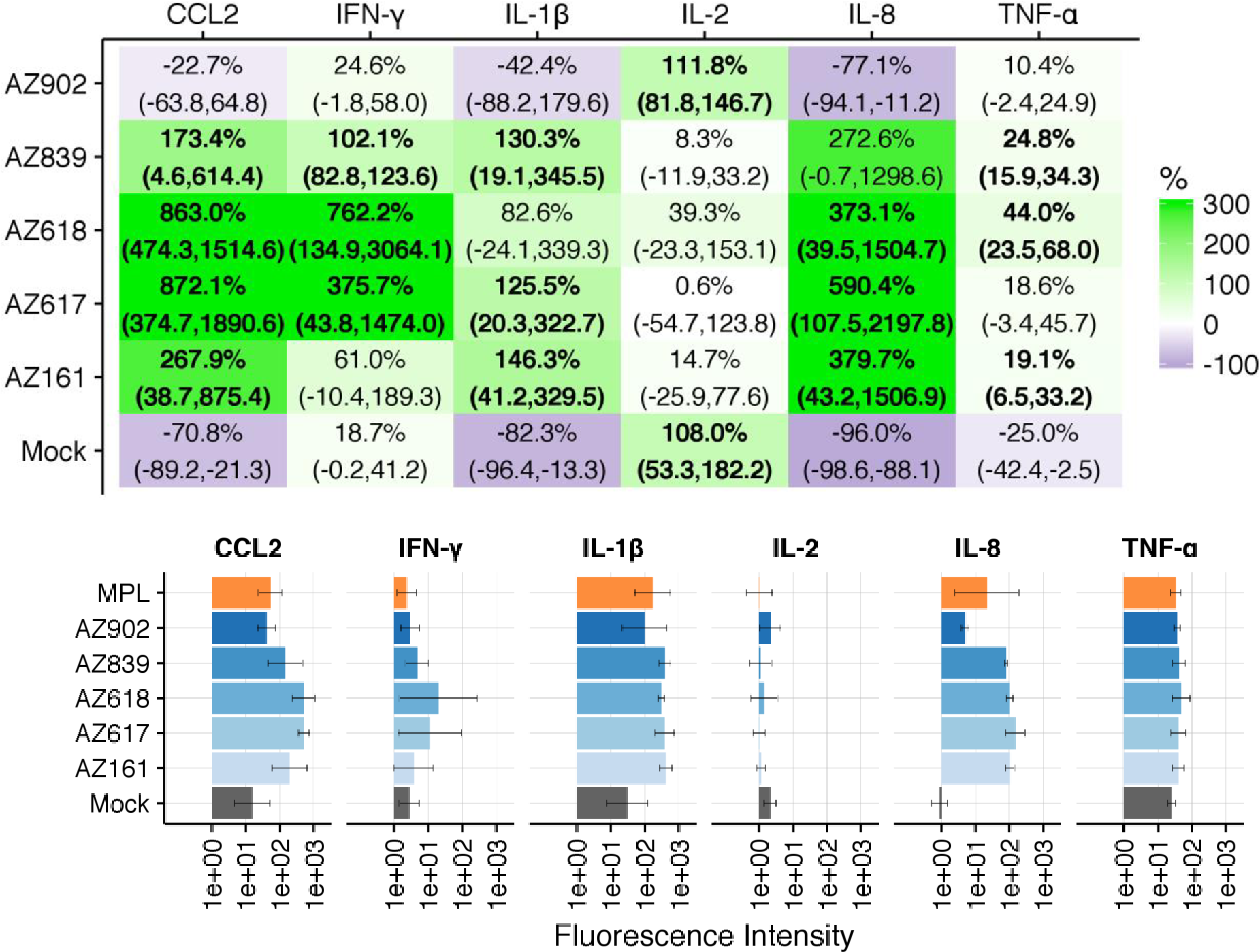

Supplement: S4 Fig — Mock refers to unstimulated cells. Upper panel: Data presented in Fig 7 are expressed as mean compound % difference from the mean MPL value for that cytokine with associated 90% confidence interval (CI) listed below. A compound is considered significantly higher than MPL if the lower bound of the CI is higher than 0% (p value < 0.05); such values are in bold. Increasing shades of green and purple indicate increasingly higher and lower respective values than values achieved by MPL stimulation. Lower panel: Data presented in Fig 7 are here represented as bar graph means with SEM bars. (TIF) [file pone.0164632.s004.tif]

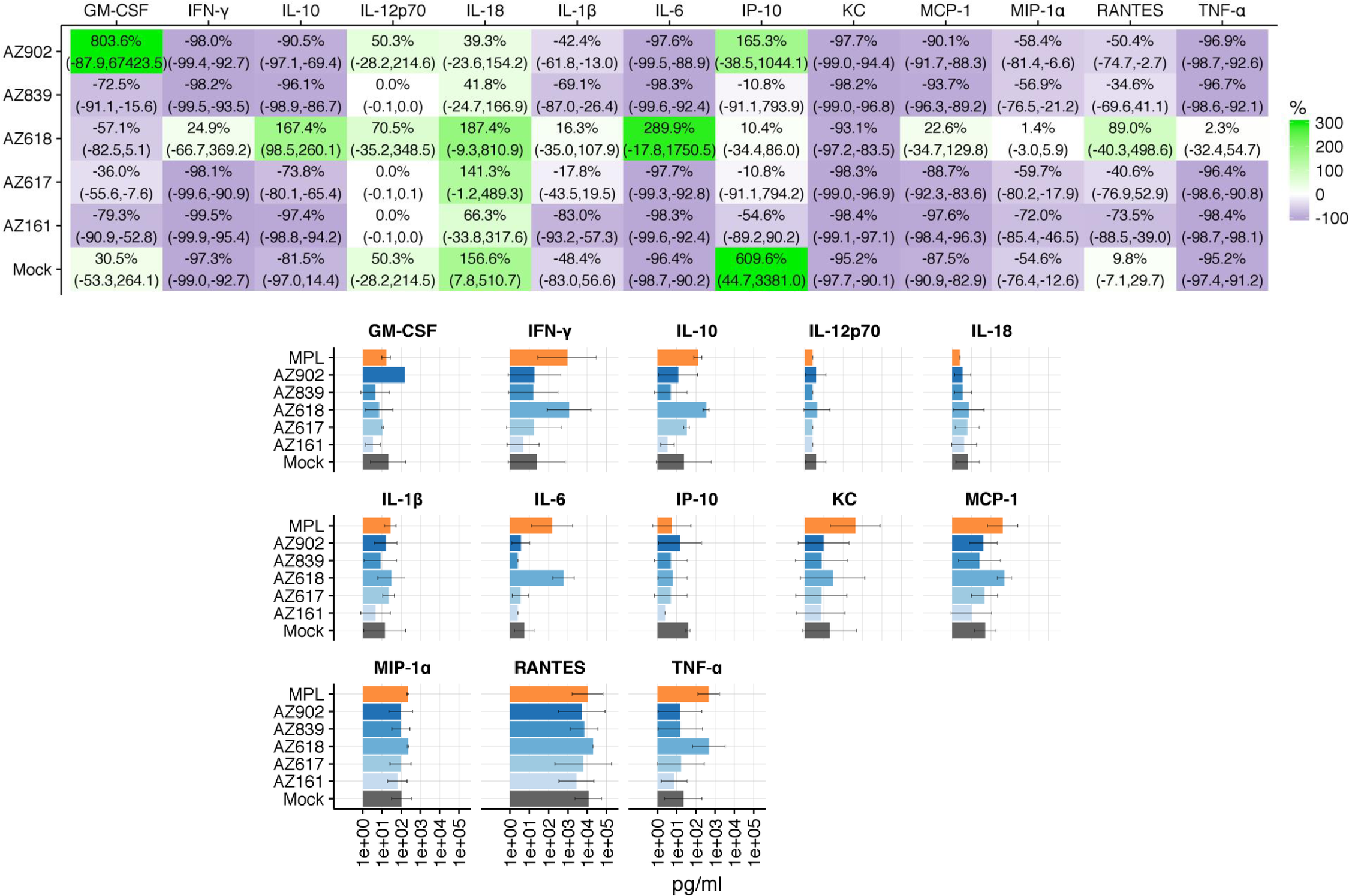

Supplement: S5 Fig — Mock refers to unstimulated cells. Upper panel: Data presented in Fig 8 are expressed as mean compound % difference from the mean MPL value for that cytokine with associated 90% confidence interval (CI) listed below. A compound is considered significantly higher than MPL if the lower bound of the CI is higher than 0% (p value < 0.05). Increasing shades of green and purple indicate increasingly higher and lower respective values than values achieved by MPL stimulation. Lower panel: Data presented in Fig 8 are here represented as bar graph means with SEM bars. (TIF) [file pone.0164632.s005.tif]

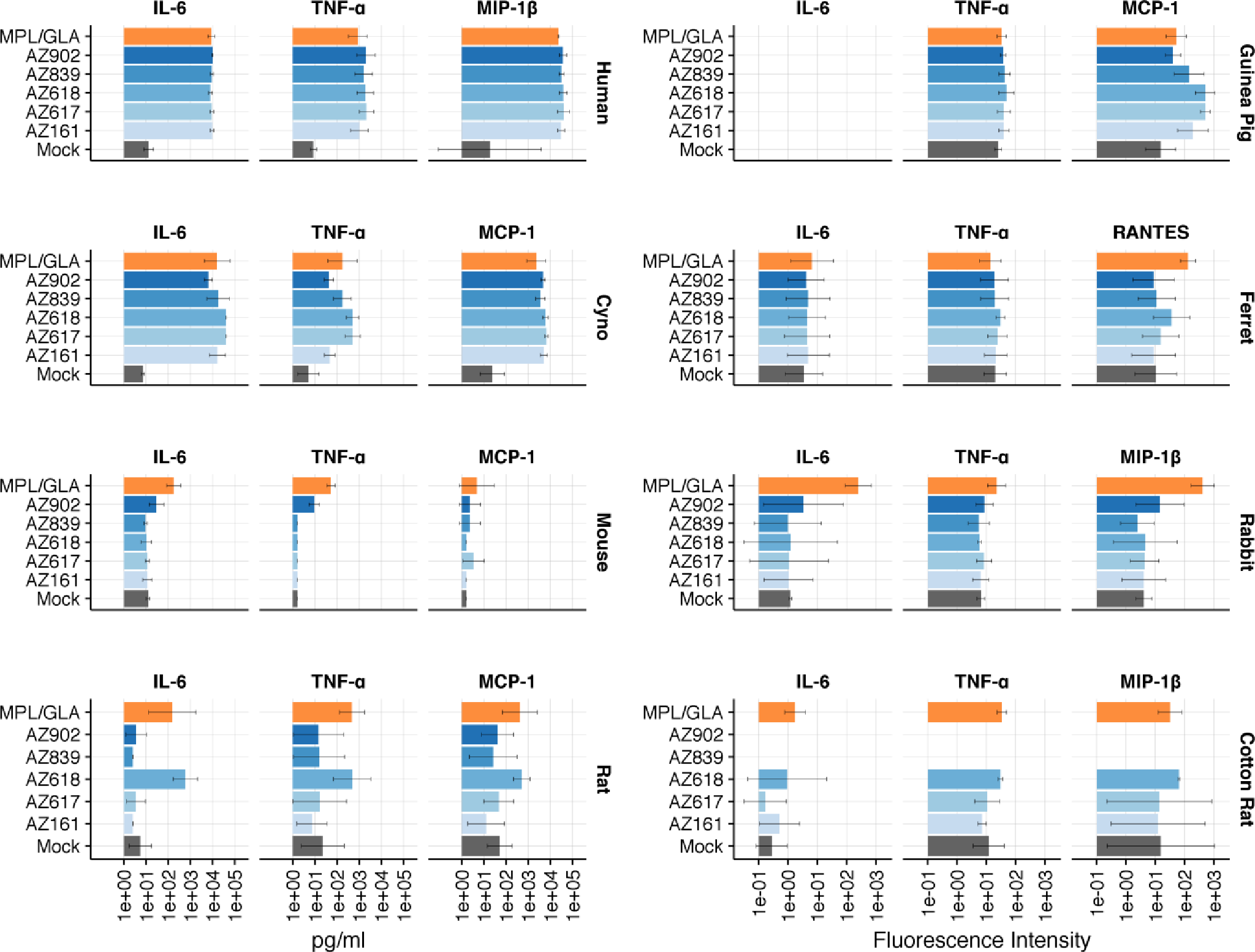

Supplement: S6 Fig — Data presented in Fig 9 here represented as bar graph means with SEM bars. Mock refers to unstimulated cells. (TIF) [file pone.0164632.s006.tif]
